# Geographic patterns in seasonal changes of body mass, skull, and brain size of common shrews

**DOI:** 10.1002/ece3.7238

**Published:** 2021-02-14

**Authors:** Javier Lázaro, Lucie Nováková, Moritz Hertel, Jan R. E. Taylor, Marion Muturi, Karol Zub, Dina K. N. Dechmann

**Affiliations:** ^1^ Max Planck Institute of Animal Behavior Radolfzell Germany; ^2^ Department of Biology University of Konstanz Konstanz Germany; ^3^ Department of Zoology Faculty of Science Charles University Prague 2 Czech Republic; ^4^ Department of Behavioural Neurobiology Max Planck Institute for Ornithology Seewiesen Germany; ^5^ Faculty of Biology University of Białystok Białystok Poland; ^6^ Mammal Research Institute Polish Academy of Sciences Białowieża Poland

**Keywords:** brain mass, Dehnel's Phenomenon, life‐stage cycling, phenotypic flexibility, seasonal plasticity, *Sorex araneus*

## Abstract

1. Some small mammals exhibit Dehnel's Phenomenon, a drastic decrease in body mass, braincase, and brain size from summer to winter, followed by a regrowth in spring. This is accompanied by a re‐organization of the brain and changes in other organs. The evolutionary link between these changes and seasonality remains unclear, although the intensity of change varies between locations as the phenomenon is thought to lead to energy savings during winter.
2. Here we explored geographic variation of the intensity of Dehnel's Phenomenon in *Sorex araneus*. We compiled literature on seasonal changes in braincase size, brain, and body mass, supplemented by our own data from Poland, Germany, and Czech Republic.
3. We analyzed the effect of geographic and climate variables on the intensity of change and patterns of brain re‐organization.
4. From summer to winter, the braincase height decreased by 13%, followed by 10% regrowth in spring. For body mass, the changes were −21%/+82%, respectively. Changes increased toward northeast. Several climate variables were correlated with these transformations, confirming a link of the intensity of the changes with environmental conditions. This relationship differed for the decrease versus regrowth, suggesting that they may have evolved under different selective pressures.
5. We found no geographic trends explaining variability in the brain mass changes although they were similar (−21%/+10%) to those of the braincase size. Underlying patterns of change in brain organization in northeastern Poland were almost identical to the pattern observed in southern Germany. This indicates that local habitat characteristics may play a more important role in determining brain structure than broad scale geographic conditions.
6. We discuss the techniques and criteria used for studying this phenomenon, as well as its potential presence in other taxa and the importance of distinguishing it from other kinds of seasonal variation.

## Graphical abstract

Some shrews seasonally shrink and regrowth their skull and brain. These changes partially match a geographic pattern which indicates that the shrinkage is more pronounced in locations with more extreme winter conditions

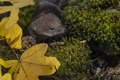

## 1 INTRODUCTION

The adaptive value of phenotypic traits can be identified only when functional correlations with environmental variables are considered. Phenotypical variation between populations and individuals is often used to address this. However, individual phenotypic flexibility, where the adult phenotype can still be modified in response to environmental change, can be hidden by this approach (Piersma & Drent, 2003). A special case of such phenotypic change is life‐stage cycling, that is, seasonal changes along the lifetime of individuals that are reversible. Studying life‐stage cycling allows the inference of mechanisms of adaptation to the environment as the changes are well marked and predictable.

**FIGURE 1:**
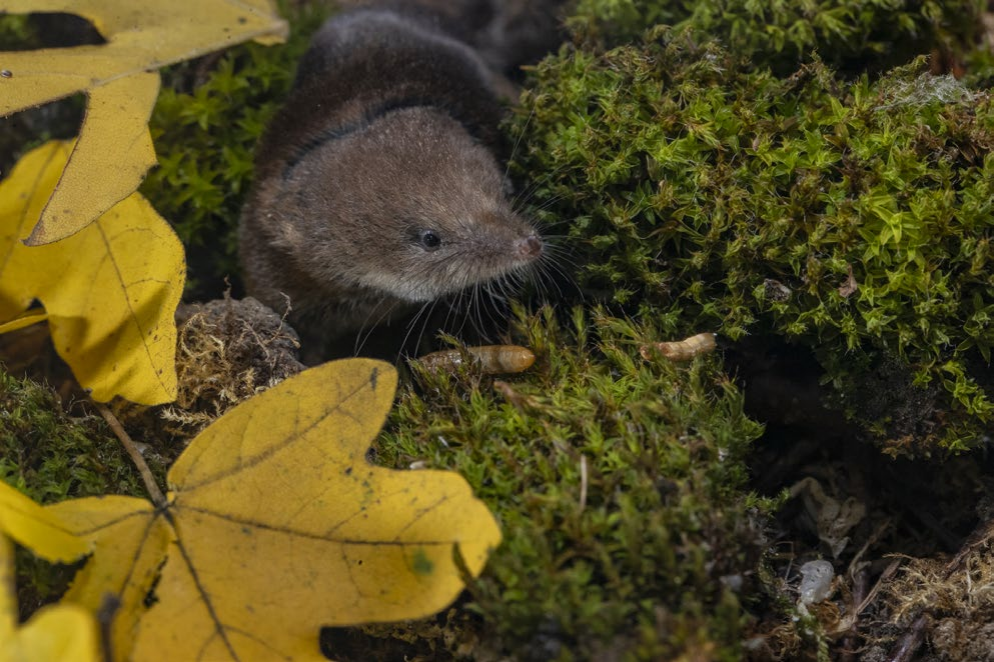
Juvenile of common shrew (*Sorex araneus*). Photo by Christian Ziegler

An outstanding case of seasonal phenotypic flexibility is the drastic but reversible morphological changes called Dehnel's Phenomenon (Dehnel, 1949), observed in some small, short‐lived mammals with high metabolic rates. In this phenomenon, young animals reach a first maximum size in their first summer, followed by a size decrease reaching a minimum size in winter. They then regrow in the spring along with sexual maturation. Best studied in the common shrew (*Sorex araneus*, Figure 1), Dehnel's Phenomenon entails a decrease in overall size, the size of the skull, and other parts of the skeleton, but also the brain and many other organs and tissues, followed by regrowth (Dehnel, 1949; Pucek, 1965). Brain mass, for example, decreases by up to 30% from summer to winter and increases again by 10%–17% during the next spring and summer (Bielak & Pucek, 1960; Lázaro, Hertel, LaPoint, et al., 2018). Braincase height, often used as a proxy for braincase size, decreases by up to 18% and regrows by up to 15% (Crowcroft & Ingles, 1959; Homolka, 1980; Yaskin, 1994). Importantly, Dehnel's Phenomenon causes not just a rescaling of the animal, but each organ and even each brain region show a unique pattern of the direction and intensity of changes, resulting in several completely different phenotypes along the year (Lázaro et al., 2018; Yaskin, 1994). Also, the length of the spine decreases and regrows seasonally as a result of shrinkage of the intervertebral disks (Hyvärinen, 1969; Saure & Hyvärinen, 1965). Some other species of shrews, and, as has recently been found, some mustelids, also show seasonal reversible shrinkage and regrowth at least of their skulls and brains (Dechmann et al., 2017; LaPoint et al., 2017).

Species known to exhibit Dehnel's Phenomenon are small short‐lived predators with very high metabolic rates, which do not hibernate or migrate during winter (Ochocińska & Taylor, 2005; Taylor, 1998). They remain active and depend on high quality food year‐round. The reversible changes of body and brain were hypothesized to be a winter adaptation to save energy and reduce resource requirement during harsh conditions (Mezhzherin, 1964; Pucek, 1970; Yaskin, 2011). While direct evidence of a link between the changes in overall size or specific organs, such as the brain and individual survival is still lacking, reducing metabolically expensive organs, including the brain during winter, is thought to decrease overall energetic needs and thus food intake (Churchfield, 1982; Schaeffer et al., 2020). Shrews use additional strategies to save energy in winter; important, for example, is increased insulation by 19% through winter fur (Taylor et al., 2013). However, this is not the complete story, as there is no seasonal overall difference in relative oxygen consumption. Winter shrews use the same amount of energy per unit of mass as summer shrews, under constant (Taylor et al., 2013) and even at ambient temperatures that differ by as much as 30°C (Schaeffer et al., 2020). The decrease in mass from summer to winter then leads to large absolute energy savings, which are not yet physiologically understood but may at least partly be due to the reduction in size of energetically expensive organs. Thus, food requirements of the size‐decreased subadult winter shrews are also lower than in the juvenile summer animals and especially in the adult individuals, whose mass doubles in the spring (Gębczyński, 1965; Schaeffer et al., 2020; Taylor et al., 2013). This would then compensate for the disadvantages of being small, such as an increasingly unfavorable volume to surface area ratio in winter (Bergmann, 1848; Yom‐Tov & Yom‐Tov, 2005). This seasonal cycle occurs in every free‐ranging individual (Lázaro et al., 2017), with very little within‐population variation within and between years, even in *ad libitum*‐fed captives, and thus clearly has a genetic basis (cite Pucek, 1964; Taylor et al., 2013). However, the intensity of the size changes is exceptionally flexible. Captive shrews differ in the intensity of seasonal change of skull size depending on capture date or when ambient temperature is manipulated (Lázaro et al., 2019).

Ambient conditions thus play an important role for Dehnel's Phenomenon, but whether they act as triggers or evolutionary drivers or both remains unclear. It has been suggested that daylength either directly or by affecting hormone levels may act as a trigger (Quay, 1984; but see Pucek, 1964) rather than temperature, as the extent of Dehnel's Phenomenon is not affected by changing weather conditions between years within populations (Taylor et al., 2013). However, temperature is also important, at least as a modulator as captive shrews kept at constant temperature cease to express the cycle (Lázaro et al., 2019). However, Dehnel's Phenomenon can differ greatly between populations. Braincase changes associated with Dehnel's Phenomenon in weasels (*Mustela erminea* and *Mustela nivalis*) vary greatly in intensity and timing between populations at different geographic locations (LaPoint et al., 2017). Previous studies on common shrews suggested a greater winter decrease in skull and body size in northeastern Europe compared to southwestern populations (Pucek, 1970; Spitzenberger, 2001). Similarly, the re‐organization of brain structure differs greatly between two populations in Radolfzell (southern Germany, Lázaro, Hertel, Sherwood, et al., 2018) and Russia (Yaskin, 1994). This increase in the extent of seasonal size change from regions with milder winter conditions to regions with harsher winter conditions supports the hypothesis that Dehnel's Phenomenon is a winter adaptation. In fact, this trend toward lower body size when facing harsh conditions also fits well to the morpho‐geographic patterns observed in *s*ome *Sorex* species, which are smaller at higher latitudes (Ochocińska & Taylor, 2003). Several small mammal species follow a “resource rule” where body size is directly predicted by resource availability (McNab, 2010). This would explain why common shrews decrease body size in winter and predict a more pronounced summer to winter size change in environments with harsher winters—that is, at high latitudes. However, a review of latitudinal differences in seasonal body mass decrease did not find any significant trend (Ochocińska & Taylor, 2003).

We compiled all published work on Dehnel's Phenomenon, discuss progress made since the last literature review in 1970 (Pucek, 1970), and take advantage of the larger currently available dataset to statistically test for the influence of geographic and climatic variables on the intensity of Dehnel's Phenomenon in *S. araneus*. We collected information from those studies which include changes in skull size and/or brain mass. From these studies, we also collected total body mass when reported and explored correlations of the intensity of Dehnel's Phenomenon with climatic and geographic variables. We added our own data on braincase size, brain mass, and body mass from new populations in Poland and body mass and braincase size from a population in the Czech Republic to this dataset. We expected to find increasing strength of Dehnel's Phenomenon along a geographic gradient of increasingly harsh seasonal environmental conditions, as predicted by previous authors. In addition, we compiled information on Dehnel's Phenomenon in other species and compared their results with *S. araneus*. Finally, we specifically investigated the variation in the structural changes within the brain associated with Dehnel's Phenomenon between populations. Here, we compared the divergent results from southern Germany (Lázaro, Hertel, Sherwood, et al., 2018) and Russia (Yaskin, 1994) with our own new data from a population in northeastern Poland, situated geographically between these two. We expected to find intermediate values of structural change that would fit into a gradual, geographic pattern in this Polish population. The aim of this review was to establish an updated framework to study the evolutionary aspects of this fascinating phenomenon.

## 2 METHODS

### 2.1 Data compilation on intensity of size changes from literature

We examined publications that report seasonal variation in skull size, brain mass in wild populations of the common shrew (*S. araneus*). From those publications, we additionally used values on total body mass when reported. As several studies did not report raw values, we extracted or calculated the percentage of decrease from the first summer size peak to the winter minimum, and the regrowth from winter to the second summer peak. We determined the first size peak as the month with the highest mean value for juveniles; the winter minimum as the month with the lowest mean value for winter subadults; and the second size peak as the month with highest mean value for adults. Summer juveniles are immature young individuals born in late spring or summer; winter subadults are immature individuals, which are ca. 6 months old; adults are individuals in spring and summer born the previous year. As *S. araneus* has a maximum life span of 13–18 months, there is no overlap of sexually mature individuals from two generations. When sample size in a given month was low, we determined the size extreme from two or more consecutive months. The amount of change was calculated as the difference between mean size extremes.

We determined coordinates and altitude for all locations. We extracted 19 bioclimatic variables from WorldClim Global Climate Data version 1.4 (Hijmans et al., 2005) and used averaged values from 1960 to 1990 (see results section for details).

Following the criteria listed above, we also compiled the same information on seasonal morphological variation in other wild mammal species. However, the low number of publications prevented any statistical analysis.

### 2.2 Skull dimension measurements from two museum collections

We included skull dimensions and body mass of *S. araneus* from two populations: Žofín, in the Novohradské hory mountain range (Czech Republic; 48.671838, 14.690402; new data) from the dry collections deposited at the Department of Zoology at the Charles University in Prague, which were collected from 1971 to 1977; and Białowieża National Park (Poland; 52.700000, 23.866667; Dechmann et al., 2017) at the Mammal Research Institute Polish Academy of Sciences collected in Białowieża National Park in 1946–1947. We measured braincase height, from the tympanic rings to the dorsal surface of the braincase, skull length, from the anteriormost projection of I^1^ to the occipital condyle, maximum braincase width, and lower mandible length, from the *alveolus dentalis* of the incisor to the coronoid process with digital calipers (±0.01 mm). We focused our analyses on braincase height as we had previously found it to change most strongly between seasons (Lázaro et al., 2017; Lázaro, Hertel, LaPoint, et al., 2018).

### 2.3 Collection of own additional data from two free‐ranging populations in Poland and Germany

We added our own data from two populations: Radolfzell, in the vicinity of Lake Constance (Germany; 47.764345, 8.997449; data published in Lázaro, Hertel, LaPoint, et al., (2018); and Gugny, in the Biebrza National Park (Poland, 53.347487, 22.589436; new data). All handling and sampling methods in Poland were approved by the Ministry of Environment (DLP‐III‐4102‐42/2607/14/MD, DLP‐III.4102.136.2016.AK).

We captured shrews with wooden live traps (PPUH A. Marcinkiewicz, Rajgród, Poland) baited with mealworms and checked at 2‐hr intervals. In Radolfzell, we trapped monthly from December 2013 to July 2016. In Gugny, we trapped at the estimated peak periods of the morphological change cycle, in February, June and July 2014, May 2015, and May 2016. Immediately after capture, shrews were weighed (±0.01 g) and carried to the laboratory where they were euthanized with anesthesia overdose (Isoflurane) and perfused transcardially with phosphate‐buffered saline (PBS) followed by 4% formaldehyde in PBS. Then, we extracted the skull measured braincase height, skull length, and braincase width as described above for museum specimens. After this, we extracted and weighed the brain (±0.001 g). As a proxy for body size, we corrected brain mass by the maxillary tooth row length (i.e., ratio brain mass/tooth row length), which does not change seasonally (Lázaro et al., 2017).

We classified individuals as summer juvenile, winter subadult, or adult based on the degree of gonadal development, capture date, and degree of tooth wear (Churchfield, 1990; Pankakoski, 1989). In adults, sex can be visually determined. We sexed immature individuals (juveniles and subadults) with a PCR‐based gonosomal sexing method (Roos, DPZ Gottingen, unpublished). For this, we extracted DNA from tail tip samples with a standard DNeasy kit (Qiagen, GmbH, Hilden).

### 2.4 Processing of brain tissue and calculation of brain region volumes

We quantified the volumes of brain regions based on 3D reconstructions of serial‐sectioned tissue (see Lázaro, Hertel, Sherwood, et al., 2018). Briefly, we separated the hemispheres sagittally weighed them (±0.001 g). We fixed them for 2 weeks in PBS/4% paraformaldehyde and then transferred them to PBS/0.1% sodium azide at 4°C for long‐term storage. We reconstructed all volumes from the left hemispheres. We immersed the hemispheres in a series of PBS/10, 20, and 30% sucrose before sectioning for cryoprotection. We cut coronal 30‐µm‐thick sections on a freezing sliding microtone (Reichert‐Jung Hn‐40). We mounted every fifth section on microscope slides and stained them with 0.5% cresyl violet. We traced the following brain regions: olfactory bulb, neocortex, rhinal and piriform cortices, caudoputamen, amygdala, nucleus accumbens, thalamus, hypothalamus, hippocampus, dentate gyrus, CA1, CA2, CA3, subiculum, and cerebellum and the total hemisphere (see Lázaro, Hertel, Sherwood, et al., (2018) for details) with the software Neurolucida (MBF Bioscience, Williston, VT, USA). We calculated the volume of each region based on the sum of the outlined areas multiplied by the section thickness and intersection distance using the Cavalieri principle in Neurolucida Explorer. All data from Radolfzell were previously published in Lázaro, Hertel, Sherwood, et al., (2018).

We accounted for shrinkage of tissue during the histological process with a correcting factor we calculated for each brain, as the quotient between the original hemisphere volume—determined by dividing the fresh hemisphere mass by the specific gravity of brain tissue (Stephan, 1960)—and the final reconstructed hemisphere volume. We also size‐corrected brain region volumes by the upper tooth row length (which does not change in length over the year) as a proxy for body size. Evolutionary studies on brain size comparisons sometimes use the brain stem or the rest of the brain as correcting factors, but as we are interested in relative changes in an individual phenomenon we corrected for the nonchanging tooth row.

### 2.5 Data analyses

#### 2.5.1 Analyses of large‐scale patterns from the literature and our own data

As not all sources reported raw measurements, we used the change of braincase height, brain mass, and body mass in our analyses. We fit four linear models using percentage of decrease and another four using percentage of regrowth as response variable and a single dependent variable: longitude, latitude, altitude, or the interaction latitude × longitude. A global model was not possible due to the limited number of populations in the analyses. We checked the model assumptions because we worked with percentage data. We also verified that the residuals were normally distributed with a Shapiro–Wilk test. We fit the same sets of linear models for brain mass and body mass. We used this same approach to analyze the geographic variation in absolute values (braincase height, brain mass, and body mass) between Dehnel stages. Finally, we fit linear models for these three response variables with each of the 19 climate variables (see Table 2).

#### 2.5.2 Detailed analyses of differences in seasonal skull dimensions and body mass between four populations of the common shrew

To assess the differences in braincase height, skull length, braincase width, brain mass, and body mass between stages of Dehnel's Phenomenon and locations, we used ANOVA for each of the five metrics. Dehnel's Phenomenon stage was treated as a factor with three levels (summer juvenile, winter subadult, adult). We first assessed the effect of sex on our models, even though in our previous work we found no significant influence of sex on the seasonal changes of these variables (Lázaro et al., 2017; Lázaro, Hertel, LaPoint, et al., 2018). For each response variable (braincase height, skull length, braincase width, brain mass, and body mass), we compared two models using ANOVA: (M1) included season, location, and sex and their interactions as explanatory variables. We removed sex from the second model (M2). We based our model selection on Akaike's information criterium (AIC) and chose M1 as final model for each metric if it had a lower AIC value, and the difference between the two models was significant. We used Tukey tests to perform pair‐wise comparisons between the factor levels of the final models.

#### 2.5.3 Analyses of seasonal variation in brain mass and brain region size

To analyze the variation in volume of brain regions between stages of Dehnel's Phenomenon, locations and sexes we used ANOVA for each brain region separately, with size‐corrected volume of the brain region as response variable and age, location, and sex and their interactions as explanatory variables. Here, we included sex in the model based on the significant effect we had previously found on the seasonal changes of some brain regions in Radolfzell (Lázaro, Hertel, Sherwood, et al., 2018). We did pair‐wise comparisons between factor levels using Tukey tests for multiple comparisons to disentangle the influence of season, geographic difference, and sexual dimorphism.

We ran all analyses in R 3.5.0 (R Core Team, 2015). We used the R package *stats* (R Core Team, 2015) to fit ANOVAs, linear models, and model comparisons. We used the package *mgcv* (Wood, 2015) for fitting generalized additive models. We created all plots with *ggplot2* (Wickham, 2009).

## 3 RESULTS AND DISCUSSION

### 3.1 Analyses of large‐scale patterns from the literature and our own data

**FIGURE 2:**
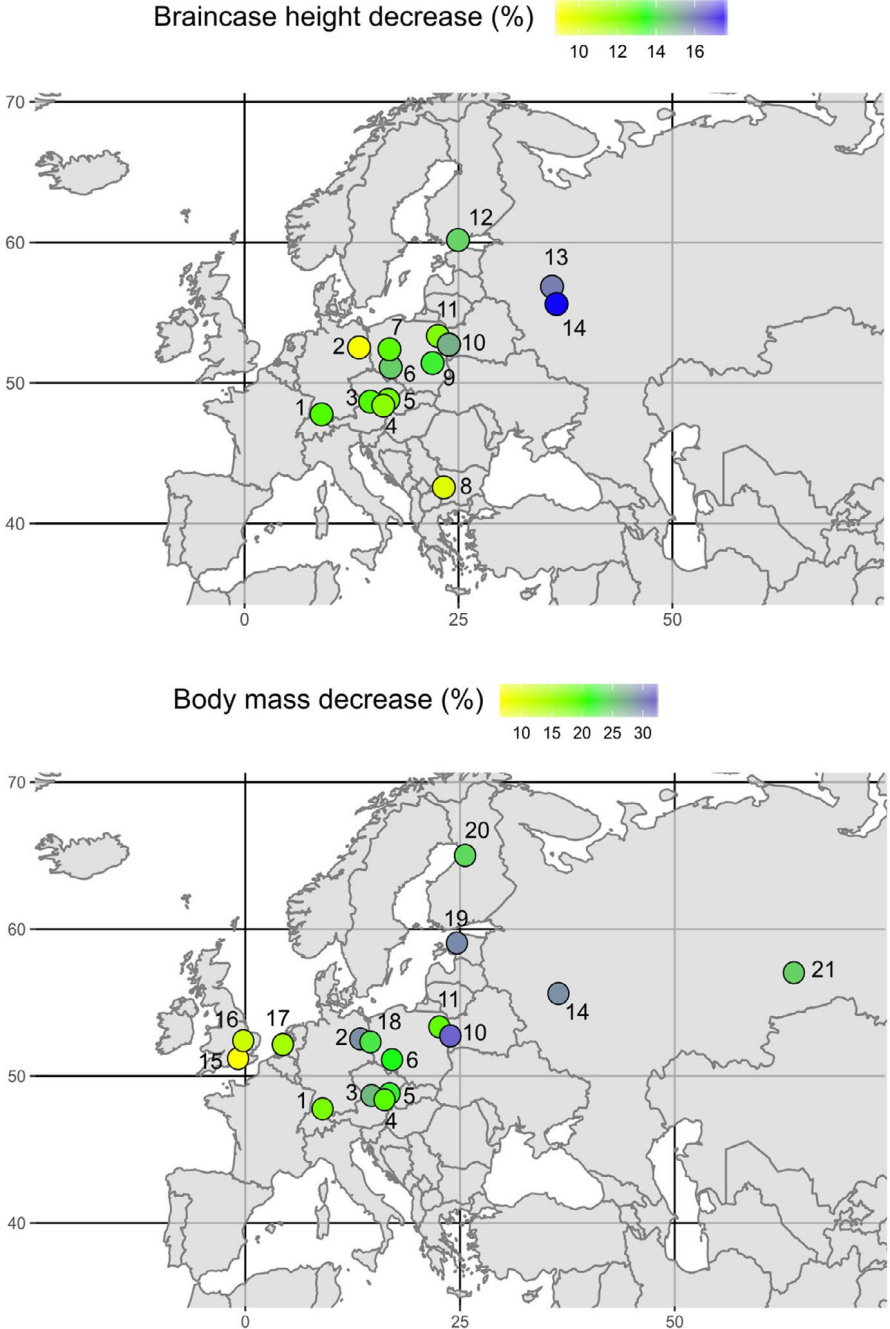
Intensity (%) of decrease in braincase height and body mass in different populations of common shrew across Europe. Each label number corresponds to a location (see Table S1): 1, Radolfzell (Ger.); 2, Berlin (Ger.); 3, Žofín (Cz.); 4, Stockerau (Aus.); 5, Lednice (Cz.); 6, Wrocław (Pol.); 7, Poznań (Pol.); 8, Vitosha (Bul.); 9, Puławy (Pol.); 10, Białowieża (Pol.); 11, Gugny (Pol.); 12, Helsinki (Fin.); 13, Tuchkovo (Russ.); 14, Moscow (Russ.); 15, Farnharm (U.K.); 16, Monks Wood (U.K.); 17, The Hague (Ned.); 18, Frankfurt (Ger.); 19, Estonia; 20, Oulu (Fin.); 21, Taliza (Russ.)

Our analyses of data from the literature review confirmed large variation in the intensity of Dehnel's Phenomenon between populations (Table S1, see reference list in Supporting Information). Mean ± *SD* decrease in braincase height from first summer peak to winter minimum was 13.4 ± 2.4%, and regrowth from winter subadults to overwintered adults was 10.3 ± 2.8%. Braincase height decrease was positively correlated with latitude, longitude, and their interaction (Figure 2, Table 1), confirming our hypotheses, but not with altitude (Table 1). Specifically, when analyzing braincase height variation across populations at each age stage, we found a negative correlation of braincase height with longitude and with the interaction of longitude and latitude at both the small subadult and regrown adult stages (*p*(sub.‐long.) < 0.05; *p*(sub.‐long.:lat.) < 0.05; *p*(ad.‐long.) < 0.05; *p*(ad.‐long.:lat.) < 0.05), but no trends along with other variables. This means that braincase height of subadults and adults, but not of juveniles, decreased toward the Northeast. The negative correlation of braincase height with longitude in subadults and adults contradicts a previous finding (Ochocińska & Taylor, 2003) of a positive correlation of condylobasal length with longitude; this indicates that there is not only a gradient in size changes, but also in skull shape between populations.

**TABLE 1:** Results from linear models to test correlation between intensity of Dehnel's Phenomenon and geographic variables

	F	SW	DF	Adj‐*R* ^2^	*p*	correlation
Brain case height decrease						
Latitude	5.6	>0.5	17	0.20	<0.05	Positive
Longitude	5.3	>0.5	17	0.19	<0.05	Positive
Lat. × Long.	6.6	>0.5	17	0.24	<0.05	Positive
Altitude	0.0	>0.1	15	0.01	>0.5	No
Brain case height regrowth						
Latitude	0.1	>0.1	14	0.03	>0.1	No
Longitude	0.4	>0.1	14	0.04	>0.5	No
Lat. × Long.	0.5	>0.1	14	0.03	>0.1	No
Altitude	2.2	>0.1	12	0.09	>0.1	No
Brain mass decrease						
Latitude	0.1	>0.5	8	0.12	>0.5	No
Longitude	0.2	>0.5	8	0.10	>0.5	No
Lat. × Long.	0.2	>0.5	8	0.10	>0.5	No
Altitude	0.5	>0.5	8	0.06	>0.1	No
Brain mass regrowth						
Latitude	2.8	>0.5	7	0.18	>0.1	No
Longitude	0.7	>0.1	7	0.03	>0.1	No
Lat. × Long.	0.7	>0.1	7	0.04	>0.1	No
Altitude	4.5	>0.5	7	0.30	>0.05	No
Body mass decrease						
Latitude	2.5	>0.5	24	0.06	>0.1	No
Longitude	10.8	>0.5	24	0.28	<0.01	Positive
Lat. × Long.	10.2	>0.5	24	0.27	<0.01	Positive
Altitude	0.7	>0.5	24	0.03	>0.1	No
Body mass regrowth						
Latitude	0.4	>0.5	18	0.03	>0.5	No
Longitude	2.6	>0.05	18	0.08	>0.1	No
Lat. × Long.	2.3	>0.05	18	0.06	>0.1	No
Altitude	0.9	>0.1	18	0.01	>0.1	No

Note

SW = P‐value from Shapiro–Wilk test for normality of residuals distribution. DF = degrees of freedom.

The intensity of the decrease of braincase height was also positively correlated with temperature seasonality, annual temperature range, and precipitation seasonality, while isothermality and mean temperature of the driest quarter were negatively correlated with it. (Table 2). The link between braincase height decrease and the intensity of seasonality grew stronger along a gradient of more continental climate. This is also supported by the correlations between the intensity of Dehnel's Phenomenon and other climate variables associated with seasonality. The size decrease begins in late summer, in anticipation of changes in climate and resource variability (which is influenced by climate). Thus, our results support the hypothesis that shrews shrink to lower their energetic needs and to prepare for lower resource availability in winter (Taylor et al., (2013) Schaeffer et al). Even more interesting then, is that we did not find a correlation between braincase height regrowth and any geographic variable (Table 1). Braincase height regrowth was only positively correlated with precipitation during the warmest quarter (Table 2). The abundance of the main prey of common shrews, the common earthworm (Churchfield et al., 2012), is highly dependent on soil humidity, and adult shrews might be able to afford larger brains under favorable conditions, that is, more rain.

**TABLE 2:** Results from linear models testing correlation between intensity of Dehnel's Phenomenon and climate variables

	F	DF	Adj‐R2	P	correlation
Brain case height decrease					
Annual mean temperature	0.2	17	0.05	>0.5	No
Mean diurnal temperature range	0.3	17	0.04	>0.5	No
Isothermality	5.4	17	0.20	<0.05	Negative
Temperature seasonality	9.0	17	0.31	<0.01	Positive
Max. Temperature of warmest month	0.9	17	0.01	>0.1	No
Min. Temperature of coldest month	3.1	17	0.11	>0.05	No
Temperature annual range	9.8	17	0.33	<0.01	Positive
Mean temperature of wettest quarter	1.5	17	0.03	>0.1	No
Mean temperature of driest quarter	24.8	17	0.57	<0.001	Negative
Mean temperature of warmest quarter	1.1	17	0.00	>0.1	No
Mean temperature of coldest quarter	2.6	17	0.08	>0.1	No
Annual precipitation	0.0	17	0.03	>0.1	No
Precipitation of wettest month	0.0	17	0.06	>0.5	No
Precipitation of driest month	1.5	17	0.03	>0.1	No
Precipitation seasonality	7.6	17	0.27	<0.05	Positive
Precipitation of wettest quarter	2.3	17	0.06	>0.5	No
Precipitation of driest quarter	2.1	17	0.06	>0.1	No
Precipitation of warmest quarter	0.1	17	0.05	>0.5	No
Precipitation of coldest quarter	2.9	17	0.10	>0.1	No
Brain case height regrowth					
Annual mean temperature	2.9	14	0.06	>0.5	No
Mean diurnal temperature range	0.2	14	0.06	>0.5	No
Isothermality	0.2	14	0.05	>0.5	No
Temperature seasonality	1.1	14	0.01	>0.1	No
Max. Temperature of warmest month	1.2	14	0.01	>0.1	No
Min. Temperature of coldest month	0.0	14	0.07	>0.5	No
Temperature annual range	1.4	14	0.03	>0.1	No
Mean temperature of wettest quarter	1.2	14	0.01	>0.1	No
Mean temperature of driest quarter	1.8	14	0.05	>0.1	No
Mean temperature of warmest quarter	1.0	14	0.00	>0.1	No
Mean temperature of coldest quarter	0.0	14	0.07	>0.5	No
Annual precipitation	1.6	14	0.04	>0.1	No
Precipitation of wettest month	3.4	14	0.14	>0.05	No
Precipitation of driest month	1.4	14	0.03	>0.1	No
Precipitation seasonality	0.1	14	0.06	>0.5	No
Precipitation of wettest quarter	3.5	14	0.14	>0.05	No
Precipitation of driest quarter	0.9	14	0.01	>0.1	No
Precipitation of warmest quarter	5.0	14	0.21	<0.05	Positive
Precipitation of coldest quarter	0.0	14	0.07	>0.5	No
Brain mass decrease					
Annual mean temperature	0.1	8	0.11	>0.5	No
Mean diurnal temperature range	0.2	8	0.09	>0.5	No
Isothermality	0.0	8	0.12	>0.5	No
Temperature seasonality	0.1	8	0.12	>0.5	No
Max. Temperature of warmest month	0.5	8	0.06	>0.5	No
Min. Temperature of coldest month	0.1	8	0.12	>0.5	No
Temperature annual range	0.1	8	0.11	>0.5	No
Mean temperature of wettest quarter	0.1	8	0.11	>0.5	No
Mean temperature of driest quarter	0.1	8	0.12	>0.5	No
Mean temperature of warmest quarter	0.1	8	0.11	>0.5	No
Mean temperature of coldest quarter	0.1	8	0.12	>0.5	No
Annual precipitation	0.1	8	0.12	>0.5	No
Precipitation of wettest month	0.6	8	0.05	>0.1	No
Precipitation of driest month	0.1	8	0.11	>0.5	No
Precipitation seasonality	0.0	8	0.12	>0.5	No
Precipitation of wettest quarter	0.2	8	0.10	>0.5	No
Precipitation of driest quarter	0.0	8	0.12	>0.5	No
Precipitation of warmest quarter	0.2	8	0.10	>0.5	No
Precipitation of coldest quarter	0.1	8	0.11	>0.5	No
Brain mass regrowth					
Annual mean temperature	1.1	7	0.02	>0.1	No
Mean diurnal temperature range	0.1	7	0.13	>0.5	No
Isothermality	1.7	7	0.08	>0.1	No
Temperature seasonality	0.9	7	0.01	>0.1	No
Max. Temperature of warmest month	1.8	7	0.09	>0.1	No
Min. Temperature of coldest month	1.0	7	0.00	>0.1	No
Temperature annual range	0.8	7	0.03	>0.1	No
Mean temperature of wettest quarter	5.2	7	0.34	>0.05	No
Mean temperature of driest quarter	1.2	7	0.03	>0.1	No
Mean temperature of warmest quarter	5.2	7	0.34	>0.05	No
Mean temperature of coldest quarter	1.0	7	0.00	>0.1	No
Annual precipitation	3.0	7	0.20	>0.1	No
Precipitation of wettest month	4.4	7	0.30	>0.05	No
Precipitation of driest month	2.1	7	0.12	>0.1	No
Precipitation seasonality	0.3	7	0.10	>0.5	No
Precipitation of wettest quarter	4.1	7	0.28	>0.05	No
Precipitation of driest quarter	2.3	7	0.14	>0.1	No
Precipitation of warmest quarter	4.1	7	0.28	>0.05	No
Precipitation of coldest quarter	2.0	7	0.11	>0.1	No
Body mass decrease					
Annual mean temperature	6.6	24	0.18	<0.05	Negative
Mean diurnal temperature range	3.6	24	0.10	>0.05	No
Isothermality	8.4	24	0.23	<0.01	Negative
Temperature seasonality	13.5	24	0.33	<0.01	Positive
Max. Temperature of warmest month	0.8	24	0.01	>0.1	No
Min. Temperature of coldest month	10.5	24	0.28	<0.01	Negative
Temperature annual range	13.7	24	0.34	<0.01	Positive
Mean temperature of wettest quarter	6.1	24	0.17	<0.05	Positive
Mean temperature of driest quarter	16.3	24	0.38	>0.001	Negative
Mean temperature of warmest quarter	0.0	24	0.04	>0.5	No
Mean temperature of coldest quarter	10.5	24	0.28	<0.01	Negative
Annual precipitation	4.0	24	0.11	>0.05	No
Precipitation of wettest month	0.1	24	0.04	>0.5	No
Precipitation of driest month	6.4	24	0.18	<0.05	Negative
Precipitation seasonality	9.0	24	0.24	<0.01	Positive
Precipitation of wettest quarter	0.4	24	0.03	>0.5	No
Precipitation of driest quarter	7.9	24	0.22	<0.01	Negative
Precipitation of warmest quarter	0.8	24	0.01	>0.1	No
Precipitation of coldest quarter	10.4	24	0.27	<0.01	Negative
Body mass regrowth					
Annual mean temperature	1.8	18	0.04	>0.1	No
Mean diurnal temperature range	0.7	18	0.02	>0.1	No
Isothermality	3.1	18	0.10	>0.05	No
Temperature seasonality	2.9	18	0.09	>0.1	No
Max. Temperature of warmest month	0.2	18	0.04	>0.5	No
Min. Temperature of coldest month	2.2	18	0.06	>0.1	No
Temperature annual range	2.4	18	0.07	>0.1	No
Mean temperature of wettest quarter	2.1	18	0.05	>0.1	No
Mean temperature of driest quarter	5.3	18	0.18	<0.05	Negative
Mean temperature of warmest quarter	0.0	18	0.06	>0.5	No
Mean temperature of coldest quarter	2.4	18	0.07	>0.1	No
Annual precipitation	0.1	18	0.05	>0.5	No
Precipitation of wettest month	1.0	18	0.00	>0.1	No
Precipitation of driest month	0.5	18	0.02	>0.1	No
Precipitation seasonality	4.7	18	0.16	<0.05	Positive
Precipitation of wettest quarter	0.5	18	0.02	>0.1	No
Precipitation of driest quarter	1.2	18	0.01	>0.1	No
Precipitation of warmest quarter	2.9	18	0.09	>0.1	No
Precipitation of coldest quarter	1.7	18	0.04	>0.1	No

It is striking that while the size of the brain and skull were largest in juveniles and only partially regrew after the winter decrease (Pucek, 1965) adult mass of the body, as well as several organs greatly exceeded those of subadults and even juveniles. Across all reviewed populations, mean body mass decreased by 21.2 ± 6.2% and regrew by 81.9 ± 18.2%. Energy expenditure, even at warm ambient temperatures, is by far the highest in the heavy adults and thus probably driven by mass (Schaeffer et al., 2020). Reproduction is the most important investment adult shrews face. In males, this entails territory expansion, territorial fights, and massive enlargement of the testes and in females the production of several large litters (Vlasák, 1996, 1998) all of which are energetically expensive and favored by large body size. Reproduction appears to be a terminal investment as most individuals die shortly after (Vlasák, 1996, 1998). Thus, the disproportional investment into mass instead of the brain during reproduction might be driven by the demands of reproduction. This would mean that decrease, and regrowth phases of Dehnel's Phenomenon have evolved under different evolutionary pressures, caused and modulated by independent factors. The decrease would mainly be determined by the physiological limits of shrews and the regrowth by reproduction. Matching these conclusions, we found no geographic pattern in juvenile and especially in adult body mass. If, in spite of high energetic costs, heavy adult mass is driven by reproduction, this may be a stronger selective force than local climates. In contrast, there was a significant negative correlation of body mass of winter subadults with latitude, longitude, and their interaction, that is, winter subadults had lower body mass toward northeastern populations (*p*(long.) < 0.05; *p*(lat.) < 0.001; *p*(long.:lat.) < 0.01).

Similar to braincase height, the extent of body mass changes varied between populations. Body mass decrease was positively correlated with longitude and with the interaction of latitude × longitude, but not with altitude or latitude alone (Figure 2, Table 1). Body mass regrowth was not correlated with most of geographic variables, matching the patterns found for the skull measures. Body mass decrease was also significantly correlated with most climate variables. In contrast, regrowth intensity was only negatively correlated with mean temperature during the driest quarter (the year quarter when precipitation is lowest) and, similar as in braincase height, positively correlated with precipitation during the warmest quarter (expressed as coefficient of variation, the more variation the more concentrated the precipitation on a period of the year; Table 2). Again, these patterns support the hypothesis that different evolutionary drivers are responsible for the decrease—shrinking as an adaptation to save energy during cold periods with low resource availability, and the increase—growing a large body size well adapted for territory defense and to maximize reproductive output especially in females.

The geographic patterns in the intensity of Dehnel's Phenomenon are in contrast to previous findings. Like Ochocińska and Taylor (2003), we did not find a correlation between body mass change and latitude, but when we added longitude this interaction became significant. This indicates that intensity of change might be more related with climate (the more continental the more intense) than with photoperiod (which is only correlated with latitude). Thus, photoperiod might not act as regulator, but may still be a trigger for the onset of the phenomenon. Taylor et al., (2013) found no difference in body mass and skull height between years within the same population despite different weather conditions. This apparent lack of flexibility in the phenomenon in combination with the lack of geographic variation in body mass changes (Ochocińska & Taylor, 2003) led to the hypothesis that Dehnel's Phenomenon might be genetically fixed and its intensity independent of external factors. This was supported by seasonal mass reduction in captive shrews fed ad libitum (Pucek, 1964). However, shrews kept captive at constant temperatures stopped exhibiting Dehnel's Phenomenon and animals taken into captivity at minimum size and kept at ambient conditions did not regrow skull or mass (Lázaro et al., 2019). This shows that even though the Phenomenon clearly has a genetic basis it can be extremely flexibly modified by ambient conditions. In combination with the more complete geographic analysis that includes both latitude and longitude, this indicates that Dehnel's phenomenon is at least partially determined by environmental factors and can adapt to the local environment.

While the changes in body mass we describe are dramatic, seasonal fluctuations in body mass are common in mammals. For example, North American beavers (*Castor canadensis*) lose 9%–12% of their body mass during autumn and winter, mainly because they metabolize their fat stores (Smith & Jenkins, 1997). These mass changes can be even more extreme in hibernating animals. Marmots can lose 32% of their body mass (Lenihan & Vuren, 1996) and hedgehogs 15%–28% (Haigh et al., 2012) mainly because of changes in fat tissues stored up in autumn. Body mass of nonhibernating small mammals such as voles and other rodents can also fluctuate strongly between seasons (Iverson & Turner, 1974; Merritt & Zegers, 2010; Zub et al., 2014). However, shrews and mustelids, the two taxa that are known to exhibit Dehnel's Phenomenon have extremely fast metabolisms with high fat turnover and little ability to store fat (Keicher, Szafranska citations). Yet, they also seasonally change body mass, and unlike the strategies outlined above, the size of their skull, brain, and most organs, too (Hyvärinen, 1969; Saure & Hyvärinen, 1965). Thus, Dehnel's Phenomenon should only be described in combination with other variables. The combination all these morphological changes of body mass, brain mass, skull size, the size of several internal organs, and spine length (Pucek, 1965) is an inherent part of the unique Dehnel's Phenomenon (see also general remarks below).

### 3.2 Detailed analyses of differences in seasonal skull dimensions and body mass between four populations of the common shrew

In our own previous work, we used maxillary tooth row length to correct for individual size variation (e.g., ratio braincase height/tooth row length) as it remained constant throughout the shrews' lifespan once summer juveniles reached the first size peak at our study site in southern Germany (Lázaro et al., 2017). As it was not possible to X‐ray specimens in the museum collections, we measured mandible length instead as a proxy for body size. However, when looking at three additional populations in addition to Radolfzell more closely (Žofín, Gugny and Białowieża), we found mandible length to vary between seasons (*df* = 186, adj. *R* ^2^ = 0.19, *F* = 5.3, *p*(seas.) < 0.05, *p*(loc.) < 0.001, *p*(seas.:loc.) > 0.1). Results for size‐corrected and absolute values did not significantly differ in Radolfzell (see results for corrected values in the: Figure S1 and Table S2). Consequently, we compared absolute values between the four populations. Similar to previous work, we found the strongest differences between locations and seasons in braincase height and will show only results for this skull measure below (see Table S2 for results on braincase width and length). We also tested for the effect of sex (AIC(M1) = −79.2, AIC(M2) = −67.8; ANOVA, *p* > 0.5), we excluded it from comparisons of skull dimensions. This is interesting, as the sexes differ in behavior and energetic pressures they are exposed to, particularly during reproduction, and some sexual dimorphism was found in the mandible morphology of adult *S. araneus* (Nováková & Vohralík, 2017).

In the final ANOVA exploring geographic patterns M2 (*df* = 200, adj. *R* ^2^ = 0.78, *F*(season) = 155.7, *F*(location) = 146.6, *F*(interaction seasons × location) = 1.3), we found a difference between seasons and locations at the factor level (*p* < 0.001 both), but not their interaction (*p* > 0.1). Braincase height values for all seasons combined were highest in Gugny, followed by Radolfzell, Žofín, and Białowieża (*p* < 0.05 in all pair‐wise comparisons, Table 3). As previously reported in the literature and in contrast to most other mammals, we found *S. araneus* to be smaller (as measured by braincase height) with increasing latitude. However, there were exceptions. For example, the two neighboring Polish populations differed more in size than Gugny (northeastern Poland) and Radolfzell (southern Germany), which were almost identical. This warrants further investigation, but indicates that sometimes local habitat structure may have stronger selective effects on size than climate or season.

**TABLE 3:** Summary of morphological changes between the stages of Dehnel's Phenomenon in the four studied populations

	Summer juvenile			Winter subadult			Spring/summer adult			Summer–winter change	Winter–adult change
	*n*	Mean ± SE	Period	*n*	Mean ± SE	Period	*n*	Mean ± SE	Period		
BCH (mm)											
Radolfzell	20	6.21 ± 0.04	June–July	10	5.46 ± 0.03	February	9	5.89 ± 0.06	May–June	−12.1%	7.9%
Gugny	8	6.46 ± 0.07	June–July	8	5.53 ± 0.06	February	7	6.09 ± 011	May–Jun	−12.2%	7.4%
Žofín	9	5.96 ± 0.04	July	27	5.22 ± 0.03	February	7	5.83 ± 0.05	August	−12.9%	11.7%
Białowieża	23	5.62 ± 0.05	June	4	4.77 ± 0.04	January–February	17	5.33 ± 0.03	June	−15.1%	11.3%
Corr. brain mass (g/mm)											
Radolfzell	12	0.035 ± 0.001	June–July	4	0.031 ± 0.002	February	9	0.032 ± 0.001	May–June	−11.4%	3.2%
Gugny	6	0.038 ± 0.001	June–July	8	0.032 ± 0.001	February	6	0.032 ± 0.001	May–June	−15.8%	0.0%
Body mass (g)											
Radolfzell	8	8.37 ± 0.14	July	7	7.15 ± 0.18	February	7	12.49 ± 0.26	May	−14.6%	74.7%
Gugny	10	7.79 ± 0.10	June–July	8	6.31 ± 0.17	February	6	10.90 ± 0.25	May	−19.0%	72.7%
Žofín	10	8.15 ± 0.21	August	26	6.02 ± 0.09	February	7	11.43 ± 0.34	August	−26.1%	89.9%

Abbreviation: BCH = braincase height.

**FIGURE 3:**
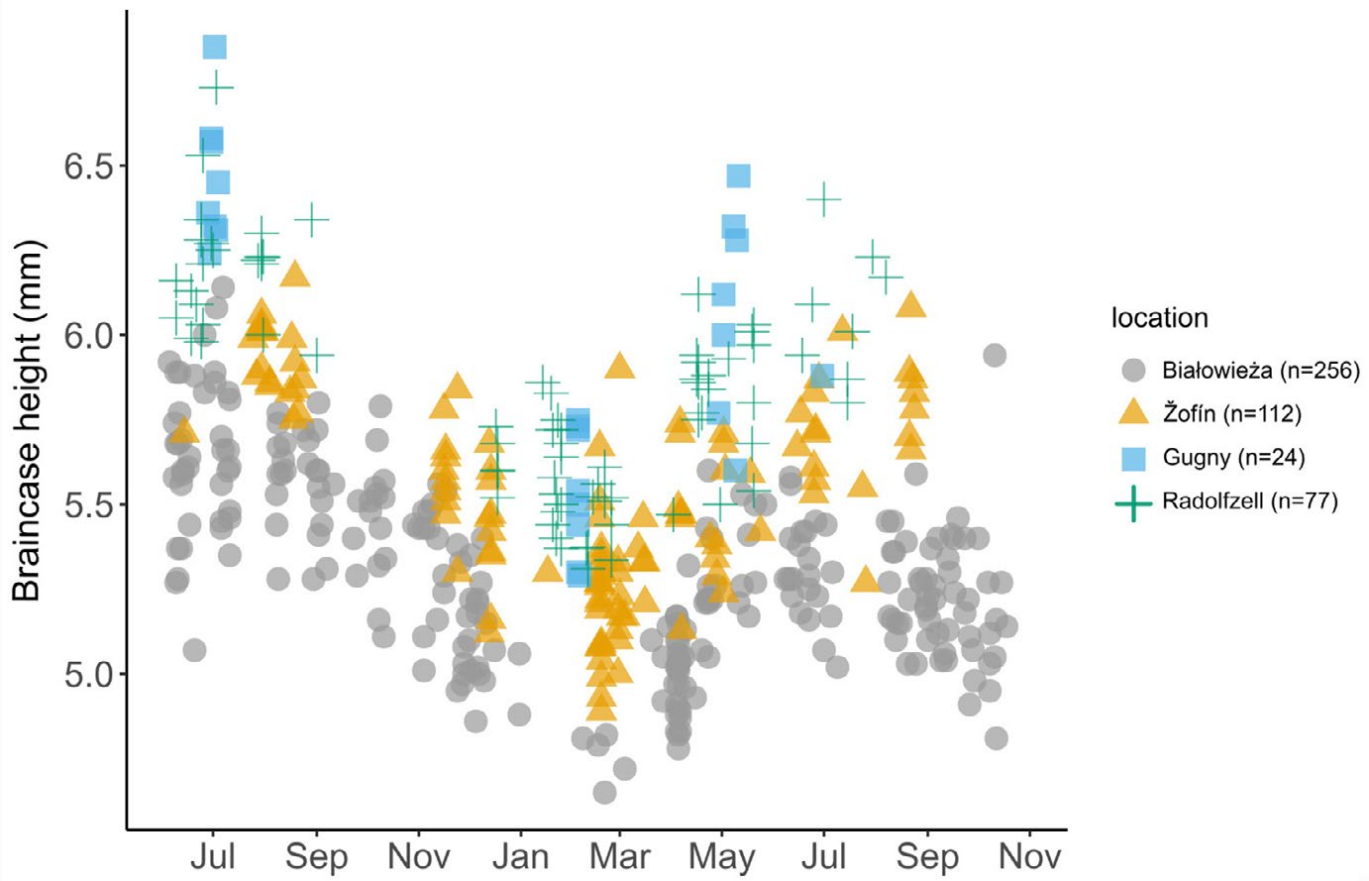
Seasonal variation in braincase height in the four populations analyzed in detail

We found that the size of shrews from the four populations differed, but not the intensity of Dehnel's Phenomenon. The Tukey post hoc test did reveal a decrease in braincase height from summer juveniles to winter subadults (*p* < 0.001) and an increase from winter subadults to adults (*p* < 0.001) and thus the presence of Dehnel's Phenomenon at all locations (Figure 3, Table 3). At a large geographic scale, we had found an increasingly strong decrease in braincase height toward northeastern populations, but also exceptions to this rule, such as strong variation in braincase height decrease between neighboring populations (e.g., within northern Germany (Schubarth, 1958)) and matching patterns in distant populations (e.g., southern Germany and central Finland (Lázaro, Hertel, LaPoint, et al., 2018; Skarén, 1964)). However, braincase height decrease in our four focal populations did not follow the north–east pattern, but they are all situated in central Europe and habitat differences may not be strong enough to result in the variation observed at a larger scale.

Similar to the results on braincase height, we found few differences in body mass between the more closely investigated populations (Radolfzell, Gugny and Žofín). After testing for an effect of sex, we again (AIC(M1) = 290.3, AIC(M2) = 293.6; ANOVA, *p* > 0.1) pooled males and females in all analyses. Body mass differed significantly between seasons and locations at both factor and interaction levels (M2, *df*=116, adj. *R* ^2^ = 0.88, *F*(seas.) = 424.2, *F*(loc.) = 13.8, *F*(seas.:loc.) = 2.8, *p*(seas.) < 0.001, *p*(loc.) > 0.001, *p*(seas.:loc.) > 0.05). Body mass of juveniles and adults was similar in all populations, but winter subadults from Žofín were lighter (*p* < 0.001). Žofín is the only high‐altitude population in our dataset.

**FIGURE 4:**
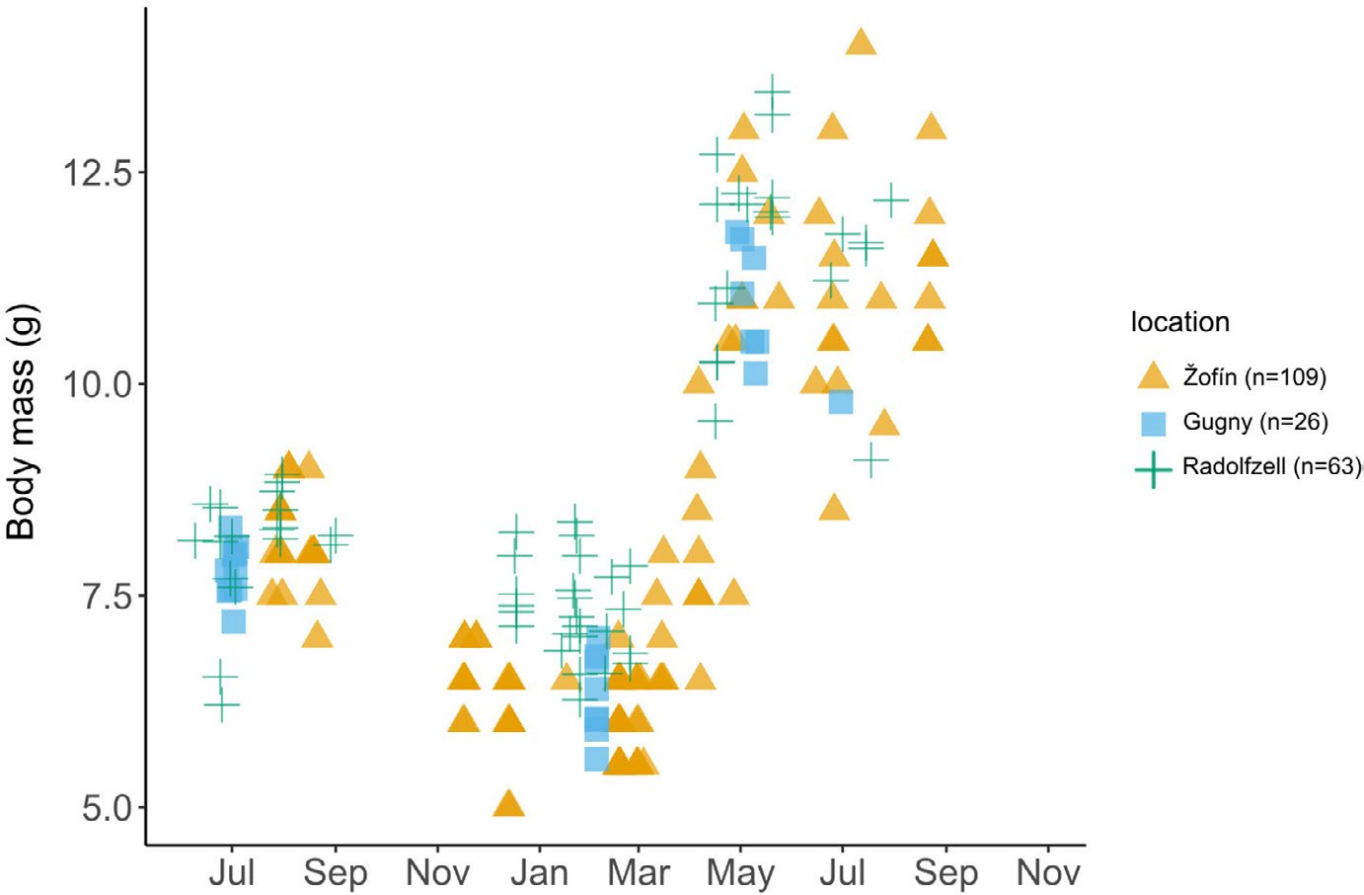
Seasonal variation in body mass in the four populations analyzed in detail

All three populations decreased mass from summer juvenile to winter subadult followed by a pronounced mass gain as they became adult (Table 3, Figure 4, Tukey test, *p* < 0.001 for all populations). Mountain populations suffer harsher winter conditions, and we expected and confirmed a stronger Dehnel's Phenomenon in shrews from Žofín. The stronger body mass decrease we found in Žofín supports the hypothesis that Dehnel's Phenomenon is a seasonal adaptation. However, we did not find a matching difference in braincase height decrease. This might mean that changes in body mass are more sensitive to local environmental differences and/or current conditions. For example, there is the little evidence for winter body mass decrease in Norway (Frafjord, 2008), but a 27% decrease was found at similar latitudes in Finland (Hyvärinen & Heikura, 1971). Alternatively, given that data from the various sites were collected during completely different years, seasonal changes in body mass may have resulted from other causes independent from Dehnel's Phenomenon, for example, winter malnutrition or nonadaptive changes. Data from Žofín are also older (1971–1977) than data from Gugny and Radolfzell (2013–2016) and differences may be linked to global warming over the last decades.

### 3.3 Analyses of seasonal variation in brain mass and brain region size

Literature on seasonal changes in mammalian brain size is scarce (see Supporting Information), but average brain mass of *S. araneus* decreased by 20.9 ± 5.6% from summer juveniles to winter subadults and regrew by 10.0 ± 4.2% to adult size (calculated with the data in Table 2). This is the most remarkable aspect of Dehnel's Phenomenon. The size of the mammalian brain, once fully grown, is usually more or less fixed and changes of this magnitude are unparalleled. Only the song center in the brain of some birds reversibly changes by similar magnitudes, but makes up only a small portion of the overall brain (Nottebohm, 1981; Tramontin et al., 1998). Experimentally induced changes in rat brain size, by starving or other environmental manipulations, result in changes of less than 5% (Bedi & Bhide, 1988). In humans, brain size increases during ontogeny, reaching a peak at the age of 20; then, after 45–50 years of age it undergoes a progressive, unidirectional decrease of 11% over the next 40 years, as a result of aging (Dekaban & Sadowsky, 1978).

**FIGURE 5:**
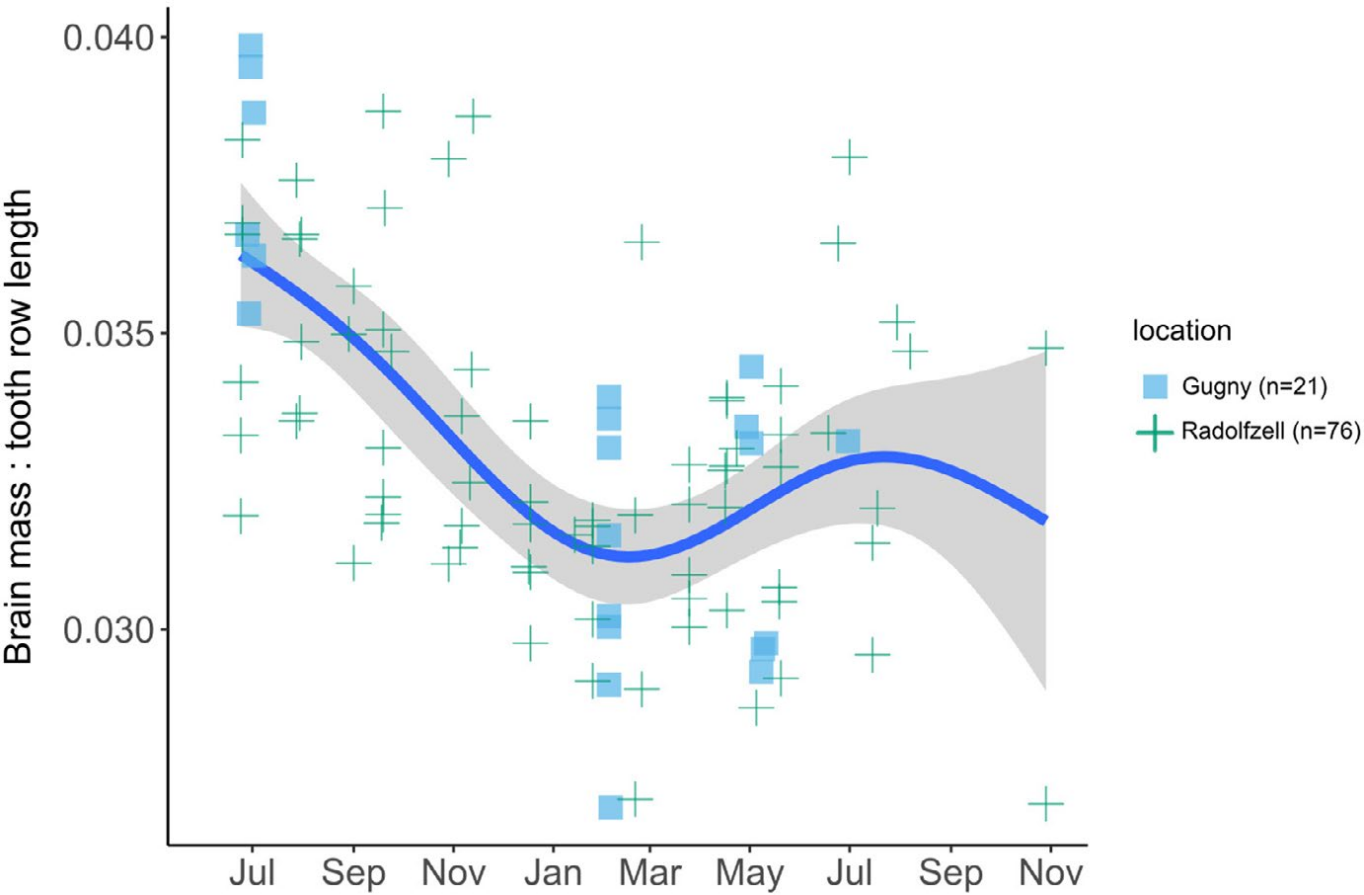
Seasonal variation in corrected brain mass in Gugny and Radolfzell with fitted Generalized Additive Model, using jday as smooth term (s), *k* = 5. Solid line and shaded area represent fitted values and standard error of the model, respectively (e.d.f. = 3.67, *F*(s) = 14, *p*(*s*) < 0.001, deviance explained = 38.4%). This fitted model helps to illustrate how adult Gugny brains were collected before the second size peak.

When we analyzed geographic variation in the intensity of seasonal brain size change in the compiled shrew literature data, we found only little variation between populations. In contrast to braincase height, we found no correlation between any geographic variable and the intensity of both decrease and regrowth of brain mass (Tables 1 and 2). When we looked at our own data from Gugny and Radolfzell in more detail, we again found no significant effect of sex on the variation of corrected brain mass (AIC(M1) = −509.6, AIC(M2) = −509.7; ANOVA, *p* > 0.1) and excluded it from the models. We found significant variation between seasons at the factor level in the final model M2, but not between locations and their interaction (*df* = 50, adj. *R* ^2^ = 0.45, *F*(seas.) = 22.5, *F*(loc.) = 0.5, *F*(seas.:loc.) = 2.1, *p*(seas.) < 0.001, *p*(loc.) > 0.5, *p*(seas.:loc.) > 0.1). There was no difference in absolute brain mass at any of the age stages that could be explained by geographic variables either (*p* > 0.1 for all models). At both locations, there was a significant but similar decrease from summer juvenile to winter subadult (Tukey test, *p* < 0.001). Surprisingly, we found no significant regrowth from winter subadult to adult (*p* > 0.1). Thus, corrected brain mass was similar at every stage in both populations (Table 3, Figure 5). This lack of spring regrowth in brain mass differs from previous research, where a significant regrowth in brain mass from winter to summer was found in all studied populations of *S. araneus* (Bielak & Pucek, 1960; Lázaro, Hertel, LaPoint, et al., 2018; Pucek, 1970; Yaskin, 1994). In fact, in our own previous study of the Radolfzell population we found a significant regrowth of brain mass, with a maximum during July–August (Lázaro, Hertel, LaPoint, et al., 2018). The adult brains used here were collected earlier (May–July). Especially in Gugny, we collected most adults in May when body mass regrowth peaks and when the regrowth peak is commonly measured. Thus, the adults we included here probably had not completed brain regrowth yet. All this emphasizes the importance of timing data collection correctly and defining the size stages for studies of Dehnel's Phenomenon carefully. An accurate estimation of the maxima and minimum of the cycle is critical for the quantification of the change. Probably most studies of Dehnel's Phenomenon do not report exact maxima and minima, and consequently, all values on change intensity presented here are timed with spring body mass maxima and underestimate the actual change in brain mass.

Striking in our combined results is the discrepancy between variables. Changes in braincase height did not match changes in brain mass, unlike in previous studies (Bielak & Pucek, 1960; Lázaro, Hertel, LaPoint, et al., 2018). The geographic patterns we found in braincase height decrease were not reflected by patterns in brain mass changes. This is probably enhanced by the small sample size of brain mass data, especially from Gugny (six adult brains). Also, only few studies (10 publications about the common shrew, the most intensively studied species) have investigated the seasonal changes in brain mass to date.

**FIGURE 6:**
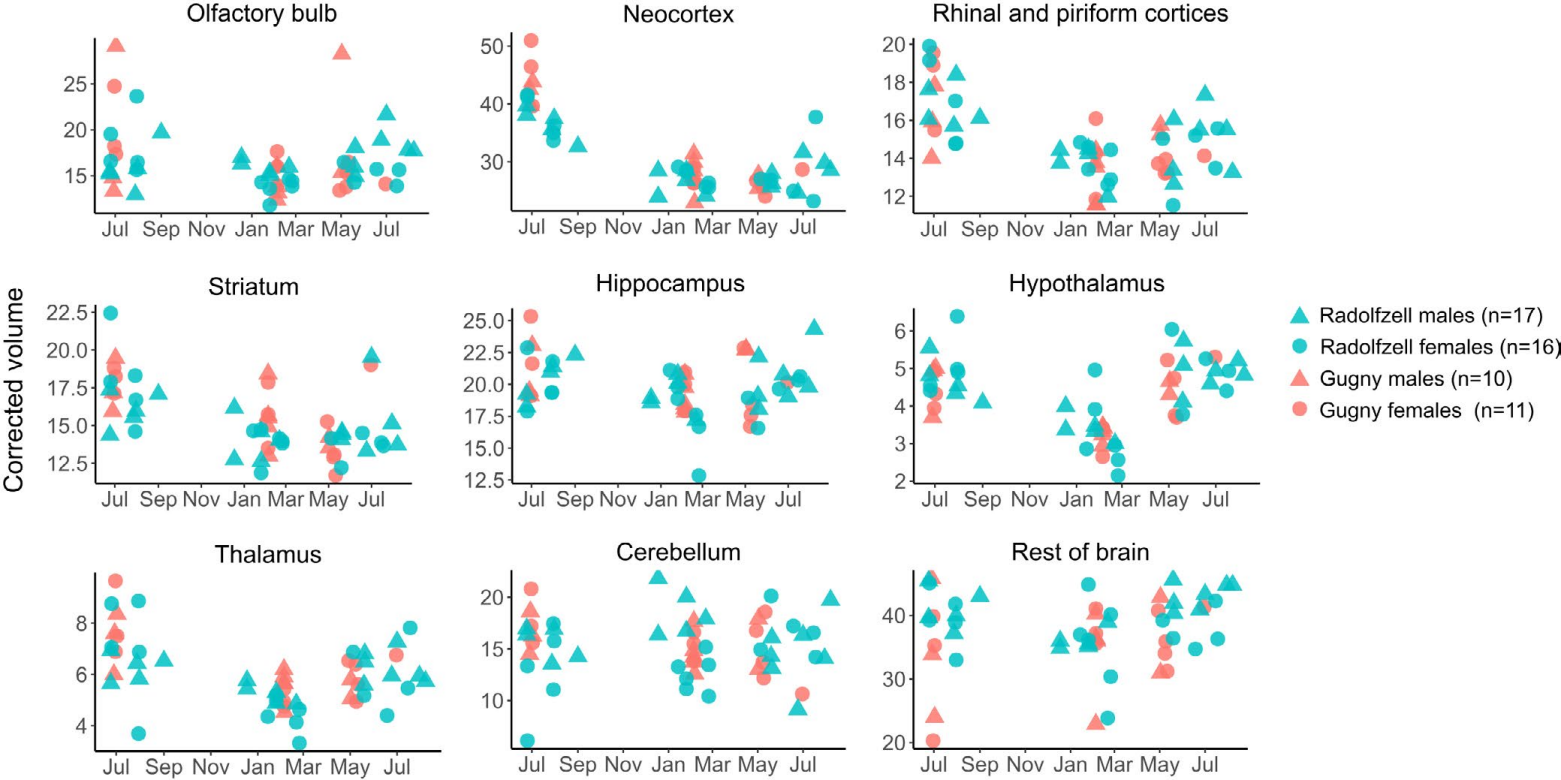
Variation between seasons and sexes in corrected volume of brain regions in Radolfzell and Gugny. The sample sizes (*n*) given are the same for each brain region. As in Figure 5, the too early collection of adult brains in Gugny is evident

The results from Gugny confirm patterns of decrease and/or regrowth (or lack of change) in the volume of each brain region from the Radolfzell population (Figure 6). Olfactory bulbs of females but not males significantly decreased from summer juveniles to winter subadults at both locations (*p* < 0.05) reflected by a significant interaction of age and sex (*F* = 4.2, *p* < 0.05), but we found no difference in seasonal olfactory bulb size between Radolfzell and Gugny at neither factor nor interaction level (*p* > 0.1). The only other brain region where we found a different pattern between the sexes was the cerebellum (see also Lázaro, Hertel, Sherwood, et al., 2018). The cerebellum did not vary seasonally at either location (*p* > 0.5). However, subadult winter females in Radolfzell had larger cerebelli than males, while in Gugny we did not find this sexual dimorphism. Changes for all other brain regions are discussed for males and females pooled and none of them differed between locations except where mentioned. Volume of the neocortex significantly decreased from summer juveniles to winter subadults (*p* < 0.001). Summer juveniles had a larger neocortex in Gugny than in Radolfzell (*p* < 0.01), but not in winter, meaning that there was a stronger decrease in Gugny. There was no difference in neocortex volumes between winter subadults and adults at either location. The rhinal and piriform cortices decreased their volume from summer to winter (*p* < 0.01) and did not regrow in adults. Also, overall striatum volume decreased from summer juveniles to winter (*p* < 0.001), but did not regrow in adults (*p* > 0.5). Within the striatum, this pattern was repeated in the caudoputamen (*p*(juv‐sub) < 0.001; *p*(sub‐ad) > 0.5) and amygdala (*p*(juv‐sub) < 0.05; *p*(sub‐ad) > 0.1), while the nucleus accumbens did not significantly change size at all. The overall volume of the hippocampus decreased from summer to winter (*p* < 0.05) and did not regrow in adults. Within the hippocampus, volume decrease was only found in CA2 (*p*(juv‐sub) < 0.05). Both the thalamus and hypothalamus decreased and regrew significantly (thalamus: *p*(juv‐sub) < 0.001; *p*(sub‐ad) < 0.05); hypothalamus: (*p*(juv‐sub) < 0.001; *p*(sub‐ad) < 0.001). The seasonal changes in all brain regions may be affected by allometric relationships, that is, the size of certain regions can be linked to the size of other region(s) (Yopak et al., 2010). Unfortunately, we could not test for allometry since we did not have enough statistical power to build such a model—which should include all brain regions as levels in an independent variable (power comparison for model based: 0.00% (0.00–0.35) based on 1,000 simulations; R package *simr,* Green & Macleod, 2016). However, we explored and mostly discarded allometric influences in these seasonal changes in the Radolfzell population (Lázaro, Hertel, Sherwood, et al., 2018).

In summary, each brain region makes a different contribution to the seasonal changes in brain size, giving rise to a marked re‐organization of the brain structure along individuals’ life. The seasonal changes in each brain region in Gugny are remarkably similar to the variation observed in Radolfzell, with the exception of a slight difference in neocortex winter decrease—more emphasized in Gugny—and a quite different pattern between males and females in the cerebellum. However, the lack of overall brain mass increase in spring in Gugny was probably be due to early sampling and the results in Lázaro, Hertel, Sherwood, et al., (2018) from Radolfzell might describe these patterns better.

Interestingly, the structural changes described from Russia (Yaskin, 1994) largely differ from both Radolfzell and Gugny. The only brain region with a similar pattern is the neocortex, which is the structure that shows the greatest winter decrease in all three populations—37% decrease in Russia and Gugny, 28% in Radolfzell—although this is followed by a 18% spring regrowth in Russia, which we did not observe in the other populations. The paleocortex of Russian shrews shrinks/regrows 28/12% in mass respectively, more pronounced than the intermediate values in the corresponding regions—rhinal and piriform cortices—in Gugny (21/6%), and the less pronounced changes in Radolfzell (18/4%). This is the only brain structure that matches our expectation of a geographic and/or environmental gradient, with the Polish population intermediate between Russia and Germany. Hippocampus changes are much greater in Russia (29/33%) than in Gugny (10/5%) and Radolfzell (10/8%), while the olfactory bulbs, which did not change seasonally in Russia, showed strong changes both in Radolfzell (14/14%) and Gugny (24/12%). These inconsistencies refute the hypothesis of a simple linear geographic trend. Instead, the differences in brain structure between populations and seasons might reflect local adaptations to specific climatic or habitat features. Nevertheless, we must also point out differences in the used methods. We used volumetric estimations derived from tracing brain regions in fixed, sectioned, and stained sections, while Yaskin (1994) weighed dissected tissue.

A recent study on the Etruscan shrew *Suncus etruscus* revealed a 3.8% decrease in cortical volume from summer to winter, and a slight regrowth in the next spring (Ray et al., 2020). The changes are most pronounced in the thickness of somatosensory cortex (28/29%). However, with this evidence we are not able yet to consider these changes as homologue to those observed in *S. araneus,* since they have been observed only in captivity, and other changes linked to Dehnel's Phenomenon as variation in skull, spine and organ size have not been studied. It would be surprising to find the Phenomenon in *Suncus,* since, despite research effort, it has never been described in white‐toothed shrews. They have much lower relative metabolic demands than *Sorex,* and they are able to enter torpor to save energy (Nagel, 1977).

### 3.4 Dehnel's Phenomenon in other species and general remarks

*Sorex araneus* is a model species for studies of Dehnel's Phenomenon. However, it is not the only species showing Dehnel's Phenomenon and, in fact, not showing the most extreme changes. We found literature on seasonal variation in braincase and/or brain size in 16 mammalian species including *S. araneus* (see the species list and data summary in Table S3). Seven of these species belong to the genus *Sorex* and 10 of them are shrews (*Soricidae*). *Sorex minutus* exhibits the most profound seasonal changes: its braincase height decreases 19.1% in winter and regrows 15.5% in spring (Kubik, 1951); and brain mass decreases up to 34.3% and regrows up to 20.3% (Caboń, 1956).

Most species showing Dehnel's Phenomenon are soricids and small mustelids. They have in common that they are small, short‐lived predators with high metabolisms, which are unable to use torpor or hibernate and which mostly delay reproduction to the spring of the year following their birth. Thus, Dehnel's Phenomenon might be a convergent adaptation to winter under similar conditions in these two phylogenetically distant groups (Dechmann et al., 2017). This is confirmed by observations of decrease in braincase and brain size in captive mustelids. Brains of captive ferrets (*Mustela putorius*) shrink by 11%–19% during 10 months after a postnatal growth peak (Apfelbach & Kruska, 1979; Weiler, 1992). A similar decrease of 14%–18% in brain mass was observed in mink from fur farms (*Mustela vison*) (Kruska, 1977) here also followed by 17% regrowth in adults (Kruska, 1993). However, we excluded these studies from our species list because the changes were not clearly linked to seasonality, and there is a known overall decreasing effect of domestication on brain size (Kruska, 1993).

There is one more taxon where seasonal size changes were observed: the morphology of arvicoline voles (Rodentia) also changes seasonally (Yaskin, 1984, 2011, 2013), even though they have a lower metabolic rate than soricids and mustelids, subsist on low quality food, and are able to reduce their metabolism in winter. And in fact, we postulate that the change in average size of skull or brain found at the population level in these species does not necessarily reflect individual size changes. Selective mortality of large individuals during summer and autumn can lead to a smaller mean body size in populations of voles and weasels in winter (Szafrańska et al., 2013; Zub et al., 2014). In contrast to shrews, which reproduce only in summer, arvicoline voles breed year‐round. Variation similar to Dehnel's Phenomenon could then be caused by seasonal size differences in cohorts, with smaller animals born in autumn and winter, as is the case in some rodents and non‐*Sorex* shrews (Brown, 1973; Dapson, 1968; Markowski & Ostbye, 1992; Schwarz et al., 1964). Confounding Dehnel's Phenomenon and a seasonal cohort effect in *Blarina brevicauda* wrongly led to the rejection of the Phenomenon (Dapson, 1968). A mean size decrease at population level can also be caused by emigration of large individuals or recruitment of small ones (Iverson & Turner, 1974). A "decrease" caused by any of these processes, might be followed by an increase in mean size, caused by the inverted process or simply by continued individual growth, which then cannot be considered a “regrowth.” Size‐corrected analyses of carefully aged individuals, such as in Dechmann et al., (2017), LaPoint et al., (2017) and Lázaro, Hertel, LaPoint, et al., (2018) are necessary to account for individual size variation and describe relative changes in the size of the brain. The only species for which Dehnel's Phenomenon in the skull and thus brain has been followed at the individual level is *S. araneus* (Lázaro et al., 2017). Mean braincase height of our southern German population in Radolfzell decreased by 12% between July and February (Lázaro, Hertel, LaPoint, et al., 2018). In that same population, recaptured individuals decreased by 15%–20% during the same period (Lázaro et al., 2017)*,* indicating that the estimations at the population level might be biased by the factors mentioned above. Thus, when studying Dehnel's Phenomenon we must carefully choose the approach and methods.

This also emphasizes that body mass should only be used in combination with other variables to describe Dehnel's Phenomenon. Individual loss in body mass from summer to winter is common and can have different causes (Zub et al., 2014). Most often it is simply a consequence of lack of resources in winter. Many species store fat resulting in a weight peak in late summer, followed by a decrease along autumn and winter as they use it up. In contrast to the anticipatory shrinking of the shrew, which also includes the skeleton and many major organs, this body mass decrease in other mammals is therefore not adaptive but a consequence of ambient conditions, which would not occur if resources were still available. Common shrews in captivity reduce food intake during winter and both body mass and braincase height decrease even when provided with food ad libitum (Churchfield, 1982; Lázaro et al., 2019). The two kinds of body mass changes—as a consequence of current ambient conditions versus adaptive—are then regulated by different physiological processes, triggered, and modulated by different external zeitgebers and are ultimately the result of different evolutionary drivers (Hyvärinen, 1984). They must be studied under separated theoretical frameworks so as not to be confounded. We suggest that individual changes in skull dimensions and brain mass are the most distinctive features of the morphological changes associated with Dehnel's Phenomenon. Until the size changes of other organs have been better described for various populations, we recommend using the extracted or scanned skull and brain in combination with body mass to verify and measure Dehnel's Phenomenon.

As important as choosing the right morphological trait to measure is the correct timing of measurements. Our brain size results from Gugny indicate that choosing the wrong timing may profoundly affect how Dehnel's Phenomenon is described in a given study. To date, the phenology of Dehnel's Phenomenon has not been investigated. To the best of our knowledge, based on our own data and the information collected from literature, the time of the year at which each stage of Dehnel's Phenomenon takes place, may vary between populations and perhaps even between years. In the common shrew, the first size peak in the summer juveniles occurs between June and August; the minimum in winter subadults has been reported between December and March; and the second peak, in sexually mature adults, is reached between May and August. The timing at each site may differ. Also, the duration of both decrease and regrowth phases has a strong impact on individuals’ biology, as it determines the rate of tissue shrinkage or regeneration. Viktorov (1967) suggested a possible geographic trend in Dehnel's Phenomenon phenology: The braincase regrowth phase tends to shorten from western (UK) to eastern (Russia) Europe, in contrast to the rate of regrowth which increases toward eastern populations. Studying the specific timing of each peak and minimum in each population might reveal correlations with current environmental factors and therefore provide more information on the triggers and evolutionary drivers of Dehnel's Phenomenon. Such added knowledge of the exact timing of the change of each tissue (bone, brain region, or organ) in conjunction with studies of gene expression and the detailed mechanisms involved will be important to truly interpret the adaptive value of Dehnel's Phenomenon. For example, the fact that the brain is largest in young dispersing juveniles and then only partially regrows in reproductive adults, which instead invest in larger body mass suggests that different drivers lead to the shrinking and the regrowth but only a detailed and holistic quantification of the costs and functions of various tissues at each stage will allow us to answer this. Perhaps then, we can understand more general questions, such as why soricine shrews and small mustelids pursue the risky strategy of reproducing only so close to the end of their brief lifespan.

## CONFLICT OF INTEREST

The authors declare no conflict of interest.

## AUTHOR CONTRIBUTIONS

**Javier Lázaro:** Conceptualization (equal); data curation (lead); formal analysis (lead); investigation (lead); methodology (equal); visualization (lead); writing – original draft (lead); writing – review and editing (lead). **Lucie Nováková:** Conceptualization (supporting); data curation (supporting); formal analysis (supporting); investigation (supporting); methodology (supporting); writing – original draft (supporting); writing – review and editing (supporting). **Moritz Hertel:** Conceptualization (equal); funding acquisition (supporting); investigation (supporting); methodology (equal); resources (equal); supervision (supporting); writing – original draft (supporting); writing – review and editing (supporting). **Jan R. E. Taylor:** Conceptualization (supporting); methodology (supporting); resources (equal); writing – original draft (supporting); writing – review and editing (supporting). **Marion Muturi:** Methodology (equal); writing – original draft (supporting). **Karol Zub:** Resources (supporting); writing – review and editing (supporting). **Dina K. N. Dechmann:** Conceptualization (equal); funding acquisition (lead); investigation (equal); methodology (supporting); project administration (lead); resources (equal); supervision (equal); validation (equal); writing – original draft (equal); writing – review and editing (equal).

## ETHICAL APPROVAL

All methods for animal handling and sampling used for this study were approved by the Polish Ministry of Environment (DLP‐III‐4102‐42/2607/14/MD, DLP‐III.4102.136.2016.AK).

## Supporting information

Supplementary Material

## Data Availability

Morphological and climate data collected from literature: Table S1. Primary data on skull and brain morphology can be found in Dryad: https://doi.org/10.5061/dryad.n2z34tmvq.
