# Supplementary material for: Geographic patterns in seasonal changes of body mass, skull, and brain size of common shrews
Source: Ecol Evol. 2021 Feb 14;11(6):2431–48. doi: 10.1002/ece3.7238 (PMC7981214; doi:10.1002/ece3.7238)
Supplement: Supplementary file 1 — Supplementary Material [file ECE3-11-2431-s001.docx]

**Supporting Information**

Geographical patterns in seasonal change in body mass, skull and brain size of common shrews

Javier Lázaro*, Lucie Nováková, Moritz Hertel, Jan R. E. Taylor, Marion Muturi, Karol Zub, Dina K. N. Dechmann

*corresponding author: [jlazaro@ab.mpg.de](mailto:jlazaro@ab.mpg.de)

**Results on skull length and braincase width variation in four populations (Radolfzell, Gugny, Žofín, Białowieża)** **of the common shrew**

Seasonal variation in skull length was less pronounced than in braincase height (see Table S2). The models comparison revealed no significant effect of sex in skull length variation (AIC(M1)= 145.0, AIC(M2)=130.3, ANOVA; *P*>0.5). In the final model M2 (d.f.=191, adj. R^2^=0.78, *F*(season)=44.3, *F*(location)=67.7, *F*(interaction seasons:location)=1.6), there was a difference between seasons and locations at the factor level (*P*<0.001 both) but not their interaction (*P*>0.1). We only found a significant decline from summer juveniles to winter subadults in Radolfzell (Tukey test, *P*<0.001); we found no other differences between seasons in any other location.

Variation in braincase width was also very low (Table S2). Differences in sex were not significant (AIC(M1)= 29.0, AIC(M2)=22.2, ANOVA; *P*>0.1). The final model M2 (d.f.=179, adj. R^2^=0.29, *F*(season)=14.9, *F*(location)=19.0, *F*(interaction seasons:location)=0.8) showed difference between seasons and locations at the factor level (*P*<0.001 both) but not their interaction (*P*>0.5). However, we only found a significant increase from winter subadults to adults in Radolfzell (Tukey test, *P*<0.05).

The results on braincase height are presented and discussed in detail in the main text. Here, we summarize the sized corrected braincase height (ratio between braincase height/mandible lenght) in Fig. S1 and Table S2.


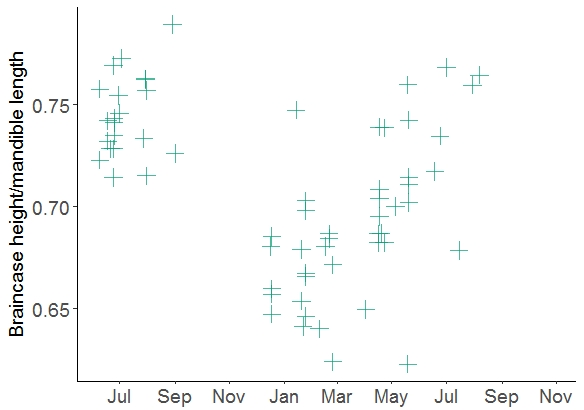


**Figure S1.** Seasonal variation in size corrected braincase height (braincase height/mandible length) in Radolfzell.

**Table S1.** Data on seasonal differences in skull and brain size in *Sorex araneus* compiled from literature, ordered by location from east to west*.* Brain and body mass are in grams, skull dimensions are in millimeters. The periods of the year when data was collected are indicated (when specified in the original source) as “t1” (summer juveniles), “t2” (winter subadults) and “t3” (spring/summer adults). Sample sizes (n) of each period are indicated as “nt1”,”nt2” and “nt3”. When percentages of decrease/increase between periods were not directly provided by the original source, they were calculated from the means of each period (“mean t1”,” mean t2”, “mean t3”). See list of references below.

| Location | Reference | Latitude | Longitude | Altitude  (m) | Metric | % decrese  t1 - t2 | % increase  t2 - t3 | nt1 | nt2 | nt3 | t1 | t2 | t3 | mean t1 | meant2 | meant3 |
| --- | --- | --- | --- | --- | --- | --- | --- | --- | --- | --- | --- | --- | --- | --- | --- | --- |
| Farnharm, UK | Churchfield 1981 | 51.188113 | -0.844145 | 119 | body mass | 6.6 | 46 | - | - | - | - | - | - | 6.46 | 6.03 | 8.93 |
| Ascot, UK | Churchfield et al. 1995 | 51.411333 | -0.642829 | 63 | body mass | 19.2 | - | - | - | - | Oct | Feb | - | - | - | - |
| Monks Wood, UK | Churchfield 1981 | 52.405456 | -0.240841 | 24 | body mass | 11.5 | 33.6 | - | - | - | - | - | - | 7.13 | 6.31 | 9.5 |
| The Hague, Netherl. | Croin Michielsen 1966 | 52.145891 | 4.368044 | 14 | body mass | 9.6 | 80.1 | 31 | 32 | 32 | Jul | Jan | - | - | - | - |
| The Hague, Netherl. | Croin Michielsen 1966 | 52.145891 | 4.368044 | 14 | body mass | 14.5 | 83.1 | 23 | 16 | 16 | Jul | Jan | - | - | - | - |
| Radolfzell, Germany | Lázaro et al. 2017 | 47.766042 | 8.997030 | 419 | body mass | 17.6 | 83 | 21 | 9 | 9 | Jul | Jan | May | 8.29 | 6.78 | 12.8 |
| Radolfzell, Germany | Lázaro et al. 2018 | 47.766042 | 8.997030 | 419 | brain mass | 21.7 | 17.6 | 6 | 4 | 4 | Jun | Feb | Jul | 0.25 | 0.20 | 0.22 |
| Radolfzell, Germany | Lázaro et al. 2018 | 47.766042 | 8.997030 | 419 | skull height | 12.9 | 13.8 | 8 | 9 | 9 | Jul | Feb | Jun-Aug | 6.27 | 5.85 | 6.22 |
| Berlin, Germany | Schubart 1958 | 52.527222 | 13.381944 | 36 | body mass | 28.92 | 96.61 | 43 | 14 | 14 | Oct | Feb | Aug | 8.3 | 5.9 | 11.6 |
| Berlin, Germany | Schubart 1958 | 52.527222 | 13.381944 | 36 | skull height | 9.17 | 7.48 | 62 | 30 | 30 | Jul | Feb | Jul | 5.89 | 5.35 | 5.75 |
| Berlin, Germany | Schubart 1958 | 52.527222 | 13.381944 | 36 | skull length | 0.59 | 1.62 | 60 | 29 | 29 | Jul | Feb | Aug | 18.63 | 18.52 | 18.82 |
| Berlin, Germany | Schubart 1958 | 52.527222 | 13.381944 | 36 | skull width | - | 3.04 | 60 | 23 | 23 | Jul | Feb | Jul | 9.17 | 9.2 | 9.48 |
| Frankfurt (Oder), Ger. | Stein 1938 | 52.317133 | 14.569189 | 73 | body mass | 22.9 | 62.8 | 41 | 17 | 17 | Oct | Feb | Apr | 8.31 | 6.41 | 10.44 |
| Žofín, Czech Republic | Present study | 48.671838 | 14.690402 | 750 | body mass | 26.13 | 89.87 | 10 | 26 | 26 | Aug | Feb | Aug | 8.15 | 6.02 | 11.43 |
| Žofín, Czech Republic | Present study | 48.671838 | 14.690402 | 750 | skull height | 12.9 | 11.7 | 8 | 27 | 27 | Jul | Feb | Aug | 5.99 | 5.22 | 5.83 |
| Bulgaria and former Czechoslovakia | Pucek 1963 | - | - | - | skull height | 9 | 7.3 | 14 | 5 | 5 | Jun | Feb | Jun | - | - | - |
| Stockerau, Austria | Spitzenberger 2001 | 48.384612 | 16.207630 | 172 | skull height | 12 | 9 | - | - | - | - | - | - | - | - | - |
| Stockerau, Austria | Spitzenberger 2001 | 48.384612 | 16.207630 | 172 | body mass | 19.4 | 68.4 | - | - | - | - | - | - | - | - | - |
| Lednice, Czech Rep. | Homolka 1980 | 48.799918 | 16.803398 | 173 | skull height | 12.4 | 14.7 | - | - | - | Jun-Jul | Feb | May | 6.05 | 5.3 | 6.08 |
| Lednice, Czech Rep. | Homolka 1980 | 48.799918 | 16.803398 | 173 | body mass | 22.35 | 95.45 | - | - | - | Jun | Jan | May | 8.25 | 6.6 | 12.9 |
| Poznań, Poland | Serafinski 1955 | 52.395000 | 16.933333 | 76 | skull height | 12.78 | 6.5 | 40 | 4 | 4 | Jun | Dec+Jan | Jul | 6.1 | 5.32 | 5.69 |
| Wrocław, Poland | Kowalska-Dyrcz 1961 | 51.116667 | 17.083333 | 155 | body mass | 21.7 | 90.6 | 21 | 5 | 5 | Jun | Jan+Feb | Jun | 7.04 | 5.51 | 10.5 |
| Wrocław, Poland | Kowalska-Dyrcz 1961 | 51.116667 | 17.083333 | 155 | brain mass | 22.1 | 11.5 | 21 | 4 | 4 | Jun | Jan+Feb | Jun | 0.21 | 0.164 | 0.182 |
| Wrocław, Poland | Kowalska-Dyrcz 1961 | 51.116667 | 17.083333 | 155 | skull height | 14.4 | 8.9 | 24 | 4 | 4 | Jun | Jan+Feb | Jun | 6.33 | 5.42 | 5.9 |
| Puławy, Poland | Kubik 1951 | 51.416550 | 21.969390 | 115 | skull height | 13.7 | 7.2 | 63 | 5 | 5 | Jul | Jan+Feb | Jul-Aug | 5.7 | 5.42 | 5.81 |
| NE Poland | Bartkowska et al. 2008 | 53.348259 | 22.592762 | 106 | brain mass | 10 | 7.9 | 16 | 17 | 17 | Jun-Aug | Dec-Feb | Jun-Aug | 0.21 | 0.19 | - |
| Gugny, Poland | Present study | 53.348259 | 22.592762 | 106 | body mass | 19 | 72.74 | 10 | 8 | 8 | Jun-Jul | Feb | May | 7.79 | 6.31 | 10.9 |
| Gugny, Poland | Present study | 53.348259 | 22.592762 | 106 | brain mass | 14.3 | - | 6 | 8 | 8 | Jun+Jul | Feb | May-Aug | 0.26 | 0.22 | - |
| Gugny, Poland | Present study | 53.348259 | 22.592762 | 106 | skull height | 12.2 | 7.4 | 6 | 8 | 8 | Jun+Jul | Feb | May+Jun | 6.46 | 5.67 | 6.09 |
| Vitosha, Bulgaria | Pucek & Markov 1964 | 42.566389 | 23.283333 | - | skull height | 9.9 | 7.08 | 29 | 28 | 28 | Jul-Aug | Jan+Feb | Jul+Aug | 5.96 | 5.37 | 5.75 |
| Vitosha, Bulgaria | Pucek & Markov 1964 | 42.566389 | 23.283333 | - | skull width | 2 | 2.76 | 48 | 17 | 17 | Jun-Oct | Nov-Feb | Mar-Oct | 9.58 | 9.41 | 9.67 |
| Białowieża, Poland | Bielak & Pucek 1960 | 52.728783 | 23.870556 | 183 | brain mass | 29.56 | 15.39 | 38 | 11 | 11 | Jul | Feb+Mar | Jul | 0.21 | 0.15 | 0.18 |
| Białowieża, Poland | Bielak & Pucek 1960 | 52.728783 | 23.870556 | 183 | braincase vol. | 26.52 | 15.73 | - | - | - | Jul | Feb | Jul | - | - | - |
| Białowieża, Poland | Bielak & Pucek 1960 | 52.728783 | 23.870556 | 183 | skull height | 12.69 | 10.34 | - | - | - | Jul | Feb | Jul | - | - | - |
| Location | Reference | Latitude | Longitude | Altitude  (m) | Metric | % decrese  t1 - t2 | % increase  t2 - t3 | nt1 | nt2 | nt3 | t1 | t2 | t3 | mean t1 | meant2 | meant3 |
| Białowieża, Poland | Dehnel 1949 | 52.728783 | 23.870556 | 183 | body mass | 24 | 102.6 | - | - | - | Sep | Jan | Aug | - | 5.7 | 11.55 |
| Białowieża, Poland | Dehnel 1949 | 52.728783 | 23.870556 | 183 | skull height | 17.67 | 12.83 | 22 | 10 | 10 | Jun | Jan+Feb | Jun | 6.34 | 5.22 | 5.89 |
| Białowieża, Poland | Pucek 1955 | 52.728783 | 23.870556 | 183 | body mass | 21.87 | 92.52 | 9 | 18 | 18 | Oct | Jan+Feb | Jul | 7.36 | 5.75 | 11.07 |
| Białowieża, Poland | Pucek 1955 | 52.728783 | 23.870556 | 183 | braincase vol. | 28.57 | 18.86 | 20 | 6 | 6 | Jun | Feb | Jun | 254.5 | 175.8 | 208.7 |
| Białowieża, Poland | Pucek 1955 | 52.728783 | 23.870556 | 183 | skull height | 15.02 | 12.03 | 19 | 6 | 6 | Jun | Feb | Jun | 6.26 | 5.32 | 5.96 |
| Białowieża, Poland | Pucek 1955 | 52.728783 | 23.870556 | 183 | skull length | 1.73 | 1.23 | 28 | 7 | 7 | Jun | Feb | Jul | 19.09 | 18.76 | 18.99 |
| Białowieża, Poland | Pucek 1955 | 52.728783 | 23.870556 | 183 | skull width | 2.23 | 3.01 | 21 | 14 | 14 | Jun | Feb | Jul | 9.86 | 9.64 | 9.93 |
| Białowieża, Poland | Pucek 1965b | 52.728783 | 23.870556 | 183 | body mass | 32.1 | 106.7 | 70 | 32 | 32 | Sep | Feb | Jun | - | - | - |
| Białowieża, Poland | Pucek 1965b | 52.728783 | 23.870556 | 183 | brain mass | 21.2 | 6.8 | 142 | 97 | 97 | Jun-Aug | Dec-Feb | May-Jul | 0.256 | 0.21 | 0.22 |
| Bialowieza, Poland | Pucek 1965a | 52.728783 | 23.870556 | 183 | brain mass | 20.9 | 5.3 | 71 | 79 | 79 | Jun-Aug | Jan-Mar | Jun-Aug | 0.263 | 0.208 | 0.219 |
| Białowieża, Poland | Taylor et al. 2013 | 52.728783 | 23.870556 | 183 | body mass | 17.8 | - | 48 | 19 | 19 | Jun+Jul | Jan+Feb | - | 7.93 | 6.52 | - |
| Białowieża, Poland | Taylor et al. 2013 | 52.728783 | 23.870556 | 183 | body mass | 18.7 | - | - | - | - | Jun+Jul | Jan+Feb | - | - | - | - |
| Białowieża, Poland | Taylor et al. 2013 | 52.728783 | 23.870556 | 183 | body mass | 20.5 | - | - | - | - | Jun+Jul | Jan+Feb | - | - | - | - |
| Białowieża, Poland | Taylor et al. 2013 | 52.728783 | 23.870556 | 183 | skull height | 12.2 | - | - | - | - | Jun+Jul | Jan+Feb | - | - | - | - |
| Białowieża, Poland | Taylor et al. 2013 | 52.728783 | 23.870556 | 183 | skull height | 14.1 | - | - | - | - | Jun+Jul | Jan+Feb | - | - | - | - |
| Estonia, various locations | Kuuse 1987 | 59.056093 | 24.642334 | - | body mass | 29.3 | 91.3 | - | - | - | Oct | Jan | Apr | 8.44 | 5.97 | 11.38 |
| Helsinki, Finland | Skarén 1964 | 60.173333 | 24.948611 | - | skull height | 14.31 | 10.06 | 13 | 6 | 6 | Jun | Jan+Feb | Jun | 6.15 | 5.27 | 5.8 |
| Oulu, Finland | Hyvarinen & Heikura 1971 | 65.017294 | 25.595138 | 15 | body mass | 26.9 | - | 44 | 30 | 30 | Sep | Jan | - | 7.1 | 5.19 | - |
| Oulu, Finland | Hyvarinen 1969 | 65.017294 | 25.595138 | 15 | body mass | 24.6 | 88.61 | 13 | 29 | 29 | Aug | Mar | Jun | 7.07 | 5.33 | 10.05 |
| Oulu, Finland | Pasanen 1971 | 65.017294 | 25.595138 | 15 | body mass | 24.1 | 89.7 | 65 | 51 | 51 | Sep | Jan | Jun | 7.05 | 5.35 | 10.15 |
| Joensuu, Finland | Hyvarinen 1994 | 62.610277 | 29.771098 | 117 | body mass | 17.9 | - | 16 | 11 | 11 | Aug+Sep | Jan+Feb | - | 6.7 | 5.5 | - |
| Kalinin, Tver, Russia | Viktorov 1967 | 56.851976 | 35.933533 | 135 | skull height | 15.98 | 10.88 | - | - | - | Jun | Feb | - | - | - | - |
| Tuchkovo, Russia | Yaskin 1994 | 55.615880 | 36.458302 | 146 | body mass | 28.94 | 89.48 | 10 | 18 | 18 | Jun-Aug | Feb+Mar | Jun-Aug | 7.36 | 5.23 | 9.91 |
| Tuchkovo, Russia | Yaskin 1994 | 55.615880 | 36.458302 | 146 | brain mass | 26.33 | 7.25 | 10 | 18 | 18 | Jun-Aug | Feb+Mar | Jun-Aug | 0.26 | 0.19 | 0.21 |
| Tuchkovo, Russia | Yaskin 1994 | 55.615880 | 36.458302 | 146 | skull height | 17.8 | 14.57 | - | - | - | Jun-Aug | Feb | Jun-Aug | 6.01 | 4.94 | 5.36 |
| Moscow, Russia | Pucek 1963 | 55.752586 | 37.590879 | 156 | skull height | 12.5 | - | - | - | - | Jun | Feb | - | - | - | - |
| Taliza, Russia | Yaskin 1994 | 57.033625 | 63.876613 | 123 | body mass | 24.5 | 73.92 | 103 | 18 | 18 | Jun-Aug | Feb+Mar | Jun-Aug | 7.06 | 5.33 | 9.27 |
| Taliza, Russia | Yaskin 1994 | 57.033625 | 63.876613 | 123 | brain mass | 19.77 | 6.76 | 103 | 18 | 18 | Jun-Aug | Feb+Mar | Jun-Aug | 0.26 | 0.21 | 0.22 |
| Taliza, Russia | Yaskin 1984 | 57.033625 | 63.876613 | 123 | brain mass | 23.6 | 11.8 | - | - | - | Jun-Aug | Feb+Mar | Jun-Aug | - | - | - |
| Former USSR | Pucek 1963 | - | - | - | skull height | 16.7 | 13.7 | 40 | 24 | 24 | Jun | Feb | Jun | 6.28 | 5.23 | 5.94 |
| Former USSR | Pucek 1963 | - | - | - | skull width | 1.8 | 2.7 | 151 | 118 | 118 | Jun-Nov | Dec-Apr | May-Sep | 9.62 | 9.45 | 9.71 |

**Table S2.** Summary of changes in skull length, braincase width and size-corrected braincase height (braincase height/mandible length) in the four studied populations.

|  | summer juvenile | | | winter subadult | | | spring/summer adult | | | summer - winter change | winter - adult change |
| --- | --- | --- | --- | --- | --- | --- | --- | --- | --- | --- | --- |
| Skull length (mm) | n | mean | period | n | mean | period | n | mean | period |  |  |
| Radolfzell | 19 | 20.42 | Jun-Jul | 24 | 19.76 | Feb | 9 | 19.96 | May-Jun | -3.2% | 1.0% |
| Gugny | 8 | 20.14 | Jun-Jul | 8 | 19.92 | Feb | 7 | 19.75 | May-Jun | -1.0% | -0.8% |
| Žofín | 9 | 19.34 | Jul | 25 | 18.99 | Feb | 7 | 19.20 | Aug | -1.8% | 1.1% |
| Bialowieza | 40 | 19.77 | Jun | 4 | 19.38 | Jan-Feb | 16 | 19.42 | Jun | -2.0% | 0.2% |
| Braincase width (mm) | | |  |  |  |  |  |  |  |  |  |
| Radolfzell | 20 | 9.60 | Jun-Jul | 24 | 9.51 | Feb | 14 | 9.81 | May-Jun | -0.9% | 3.2% |
| Gugny | 8 | 9.62 | Jun-Jul | 8 | 9.39 | Feb | 7 | 9.72 | May-Jun | -2.4% | 3.5% |
| Žofín | 8 | 9.19 | Jul | 27 | 9.18 | Feb | 17 | 9.38 | Aug | -0.1% | 2.1% |
| Bialowieza | 35 | 9.51 | Jun | 3 | 9.46 | Jan-Feb | 20 | 9.61 | Jun | -0.5% | 1.6% |
| Corr. Braincase height | |  |  |  |  |  |  |  |  |  |  |
| Radolfzell | 20 | 0.89 | Jun-Jul | 10 | 0.78 | Feb | 9 | 0.84 | May-Jun | -12.4% | 7.7% |

**Table S3.** Compilation of studies reporting seasonal changes in skull and/or brain size in other species different than *S. araneus.*

| species | location | measurement | summer - winter change | winter - adult change | reference |
| --- | --- | --- | --- | --- | --- |
| *Blarina brevicauda* | Ithaca, US | skull height | -6.14 | 4.26 | Dapson 1968 |
| *Clethrionomys rutilus* | Pyshma River, Taliza, Russia | brain mass | -10.3 | 19.6 | Yaskin 1984 |
| *Crocidura suaveolens* | Askania Nova, Ukraine | skull height | -10.12 | 4.95 | Mezhzherin 1988 |
| *Microtus gregalis* | Pyshma River, Taliza, Russia | brain mass | -12.7 | 26.5 | Yaskin 1984 |
| *Microtus oeconomus* | Pyshma River, Taliza, Russia | brain mass | -10.8 | 26.7 | Yaskin 1984 |
| *Mustela erminea* | Several locations | skull height | -16.8(f) -14.3 (m) |  | LaPoint et al. 2017 |
| *Mustela nivalis* | Several locations | skull height | -12.5(f) -7.3 (m) |  | LaPoint et al. 2017 |
| *Mustela nivalis* | Bialowieza, Poland | skull height (corrected by CBL) | -15.5 | 8.3 (m) | Dechmann et al. 2017 |
| *Myodes glareolus* | Pyshma River, Taliza, Russia | brain mass | -8.7 | 13 | Yaskin 1984 |
| *Neomys fodiens* | Poznan, Poland | skull height | -2.3 |  | Kardynia & Rychlik 2011 |
| *Sorex arcticus* | Yamal peninsula, Russia | skull height | -8.5 |  | Pucek 1963 |
| *Sorex caecutiens* | Former USSR (various locations) | skull height | -15.9 |  | Pucek 1963 |
| *Sorex longirotris* | Alabama and Georgia, US | skull height | -4.73 |  | French 1980 |
| *Sorex minutus* | Gugny, Poland | brain mass | -17 | 7.65 | Bartkowska et al. 2008 |
| *Sorex minutus* | Bialowieza, Poland | skull height | -14.79 | 14.56 | Caboń 1956 |
| *Sorex minutus* | Bialowieza, Poland | braincase capacity | -30.04 | 21.41 | Caboń 1956 |
| *Sorex minutus* | Bialowieza, Poland | brain mass | -34.29 | 20.35 | Caboń 1956 |
| *Sorex minutus* | Bialowieza, Poland | skull height | -18.11 | 11.78 | Dehnel 1949 |
| *Sorex minutus* | Puławy, Poland | skull height | -19.1 | 15.5 | Kubik 1951 |
| *Sorex minutus* | Pyshma River, Taliza, Russia | brain mass | -31.7 | 21 | Yaskin 1984 |
| *Sorex unguiculatus* | Former USSR | skull height | -13.7 | 11.4 | Pucek 1963 |
| *Sorex vagrans* | Maple Ridge, Vancouver, Canada | skull height | -8.7 |  | Hawes 1969 |

**References cited in Tables S1 and S3**

Bartkowska K, Djavadian RL, Taylor JRE, Turlejski K (2008) Generation recruitment and death of brain cells throughout the life cycle of *Sorex shrews* (Lipotyphla). *The European Journal of Neuroscience* 27: 1710–21.

Bielak T, Pucek Z (1960) Seasonal changes in the brain weight of the common shrew (*Sorex araneus* Linnaeus: 1758). *Acta Theriologica* 13: 297–300.

Caboń K (1956) Untersuchungen über die saisonale Veränderlichkeit der Gehirnes bei der kleinen Spitsmaus (*Sorex minutus minutus* L.). *Annales of the University of Marie Curie-Skłodowska, Section C* 10: 93–105.

Churchfield S (1981) Water and fat contents of British shrews and their role in the seasonal changes in body weight. *Journal of Zoology* 194: 165–173.

Churchfield S, Hollier J, Brown VK (1995) Population dynamics and survivorship patterns in the common shrew *Sorex araneus* in southern England. *Acta Theriologica* 40: 53–68.

Croin Michielsen N (1966) Intraspecific and interespecific competition in the shrews *Sorex araneus* L. and *S. minutus* L. *Archives Neerlandaises de Zoologie* 17: 73–174.

Dapson RW (1968) Growth patterns in a post-juvenile population of short-tailed shrews (*Blarina brevicauda*). *American Midland Naturalist* 79: 118–129.

Dechmann DKN, LaPoint S, Dullin C, Hertel M, Taylor JRE, Zub K, Wikelski M (2017) Profound seasonal shrinking and regrowth of the ossified braincase in phylogenetically distant mammals with similar life histories. *Scientific Reports* 7: 42443.

Dehnel A (1949) Studies on the genus *Sorex* L. *Annales of the University of Marie Curie-Skłodowska, Section C* 4: 17–102.

French TW (1980) Natural history of the southeastern shrew, *Sorex longirostris* Bachman. *American Midland Naturalist* 104: 13–31.

Hawes ML (1969) Ecological Adaptations in two Species of Shrews. Thesis, University of British Columbia.

Homolka M (1980) Biometrischer Vergleich zweier Populationen *Sorex araneus*. *Acta Scientiarum Naturalium Academiae Scientiarum Bohemicae Brno* 14: 1–34.

Hyvärinen H (1969) On the seasonal changes in the skeleton of the common shrew (*Sorex araneus* L.) and their physiological background. *Aquilo (Series Zoologica)* 7: 2–32.

Hyvärinen H (1994) Brown fat and the wintering of shrews. In: Merritt JF, Kirkland GL, Rose RK (eds) *Advances in the biology of shrews*, 259–269. Carnegie Museum of Natural History Special Publication 18.

Hyvärinen H, Heikura K (1971) Effects of age and seasonal rhythm on the growth patterns of some small mammals in Finland and in Kirkenes, Norway. *Journal of Zoology* 165: 545–556.

Kardynia P, Rychlik L (2011) Wintering adaptations of the pygmy shrew *Sorex minutus* and the Eurasian water shrew *Neomys fodiens*. In: Denys C (ed) *6th European Congress of Mammalogy*, 121–122. Université Pierre et Marie Curie, Muséum National d’Histoire Naturelle, Paris.

Kowalska-Dyrcz A (1961) Seasonal variations in *Sorex araneus* Linnaeus 1758 in Poland. *Acta Theriologica* 4: 268–273.

Kubik J (1951) Analysis of the Puławy population of *Sorex araneus araneus* L. and *Sorex minutus minutus* L. *Annales of the University of Marie Curie-Skłodowska, Section C* 11: 335–372.

Kuuse S (1987) The variability of the morphological features of shrews (*Sorex*, Insectivora) in the Estonian SSR. *Acta et Commentationea Universitatia Tartuensia* 15: 13–32.

LaPoint S, Keicher L, Wikelski M, Zub K, Dechmann DKN (2017) Growth overshoot and seasonal size changes in the skulls of two weasel species. *Open Science* 4: 160947.

Lázaro J, Dechmann DKN, LaPoint S, Wikelski M, Hertel M (2017) Profound reversible seasonal changes of individual skull size in a mammal. *Current Biology* 27: R1106–R1107.

Lázaro J, Hertel M, LaPoint S, Wikelski M, Stiehler M, Dechmann DKN (2018a) Cognitive skills of common shrews (*Sorex araneus*) vary with seasonal changes in skull size and brain mass. *Journal of Experimental Biology* 221.

Lázaro J, Hertel M, Sherwood CC, Muturi · M, Dechmann DKN (2018b) Profound seasonal changes in brain size and architecture in the common shrew. *Brain Structure and Function* 223: 2823–2840.

Mezhzherin VA (1988) Seasonal and age related changes in the skull and body weight of *Crocidura suaveolens*. *Vestnik Zoologii* 3: 36–40.

Pasanen S (1971) Seasonal variations in interscapular brown fat in three species of small mammals wintering in an active state. *Aquilo Series Zoologica* 11: 1–32.

Pucek Z (1955) Untersuchungen über die Veränderlichkeit des Schädels im lebenszyklus von *Sorex araneus araneus* L. *Annales of the University of Marie Curie-Skłodowska, Section C* 9: 163–211.

Pucek Z (1963) Seasonal changes in the braincase of some representatives of the genus *Sorex* from the Paleartic. *Journal of Mammalogy* 44: 523–536.

Pucek M (1965a) Water contents and seasonal changes of the brain-weight in shrews. *Acta Theriologica* 10: 353–367.

Pucek Z (1965b) Seasonal and age changes in the weight of internal organs of shrews. *Acta Theriologica* 10: 369–438.

Pucek Z, Markov G (1964) Changes in the skull of the common shrew from Bulgaria. *Acta Theriologica* 9: 363–366.

Schubarth H (1958) Zur Variabilität von *Sorex araneus araneus* L. *Acta Theriologica* 2: 175–202.

Serafinski W (1955) Morphological and ecological investigations on Polish species of the genus *Sorex* L. (Insectivora, Soricidae). *Acta Oecologica* 1: 27–86.

Skarén U (1964) Variations in two shrews, *Sorex unguiculatus* Dobson and *S. a. araneus* L. *Annales Zoologici Fennici* 1: 94–124.

Spitzenberger F (2001) *Die Säugetierfauna Österreichs*. Austria Medien-Service.

Stein GHW (1938) Biologische Studien an deutschen Kleinsäugern. *Archiv für Naturgeschichte, N.F* 7: 477–513.

Taylor JRE, Rychlik L, Churchfield S (2013) Winter reduction in body mass in a very small, nonhibernating mammal: consequences for heat loss and metabolic rates. *Physiological and Biochemical Zoology* 86: 9–18.

Viktorov L V. (1967) Geografičeskije osobennosti “javlenija Dehnelja’’ u zemlerojek Evropy. *Bull. Mosc. Obschch. Ispyt. Prir.* 72: 159–160.

Yaskin, V. A. (1984) Seasonal changes in brain morphology in small mammals. In *Winter Ecology of Small Mammals* (ed J.F. MERRIT), pp. 183-191. Carnegie Museum of Natural History Special Publication **10**, Pittsburgh.

Yaskin, V.A. (1994) Variation in brain morphology of the common shrew. In *Advances in the Biology of Shrews* (eds J.F. Merrit, G.L. Kirkland & R.K. Rose), pp. 155–161. Carnegie Museum of Natural History Special Publication 18, Pittsburgh.
